# L-asparaginase reversibly suppresses CD40-mediated B cell activation and antigen-presenting function by metabolic restriction

**DOI:** 10.1016/j.isci.2026.117144

**Published:** 2026-08-11

**Authors:** Amar Hadzic, María Alejandra García-Márquez, Pol Bannasch, Nina Haindl, Katharina Frey, Martin Kirmaier, Maximilian A. Funk, Anneli Tischmacher, Jingke Tu, Adriano Carboniero, Ludovica Vona, Sabine Oganesian, Lena Muschik, Hans Schlößer, Martin Vaeth, Werner Schmitz, Michael von Bergwelt-Baildon, David M. Cordas dos Santos, Sebastian Theurich

**Affiliations:** 1 Cancer and Immunometabolism Research Group, Gene Center LMU, Ludwig-Maximilians-Universität München, Munich, Germany; 2 Department of Medicine III, LMU University Hospital, Ludwig-Maximilians-Universität München, Munich, Germany; 3 German Cancer Consortium (DKTK), Munich Site, and German Cancer Research Center, Heidelberg, Germany; 4 Bavarian Cancer Research Center (BZKF), LMU University Hospital, Munich, Germany; 5 Center for Molecular Medicine Cologne, University of Cologne, Cologne, Germany; 6 Department of General, Visceral, Cancer and Transplantation Surgery, University of Cologne, Medical Faculty and University Hospital Cologne, Cologne, Germany. Center for Molecular Medicine Cologne (CMMC), University of Cologne, Medical Faculty and University Hospital Cologne, Cologne, Germany; 7 Würzburg Institute of Systems Immunology, Max Planck Research Group, Julius-Maximilians University of Würzburg, Würzburg, Germany; 8 Department of Biochemistry and Molecular Biology, Theodor Boveri Institute, Biocenter, Julius-Maximilians University of Würzburg, Würzburg, Germany

**Keywords:** B cells, Asparaginase, immunomodulation, Antigen-presentation, B regulatory cells

## Abstract

Metabolic reprogramming and nutrient uptake are essential for immune cell activation and adaptation. While asparagine is normally a non-essential amino acid, extracellular depletion by L-asparaginase (ASNase) is widely used to treat acute lymphoblastic leukemia and lymphoma. However, its effects on non-malignant B cells remain poorly understood. Using a model of CD40-mediated human B cell activation, we investigated the impact of ASNase on primary human B cells. Even at low, clinically sub-therapeutic concentrations, ASNase induced profound metabolic alterations, reducing glycolytic and mitochondrial respiratory capacity. This was accompanied by impaired proliferation and cluster formation without increasing cell death. Instead, ASNase suppressed B cell activation and antigen-presenting cell (APC) function while promoting the emergence of regulatory B cell phenotypes and immunosuppressive cytokine expression in a subset of cells. Supplementation with asparagine or glutamine restored APC function and proliferation, with glutamine showing slightly greater efficacy. These findings suggest that ASNase reversibly suppresses pro-inflammatory B cell functions through extensive metabolic reprogramming and may warrant evaluation as a potential immunosuppressive agent.

## Highlights

• ASNase suppresses CD40-B cell activation at sub-therapeutic concentrations
• ASNase impairs B cell proliferation without increasing cell death
• ASNase reduces APC function and promotes regulatory B cell features
• Asparagine or glutamine restore B cell function and proliferation

## Graphical abstract

**Figure:**
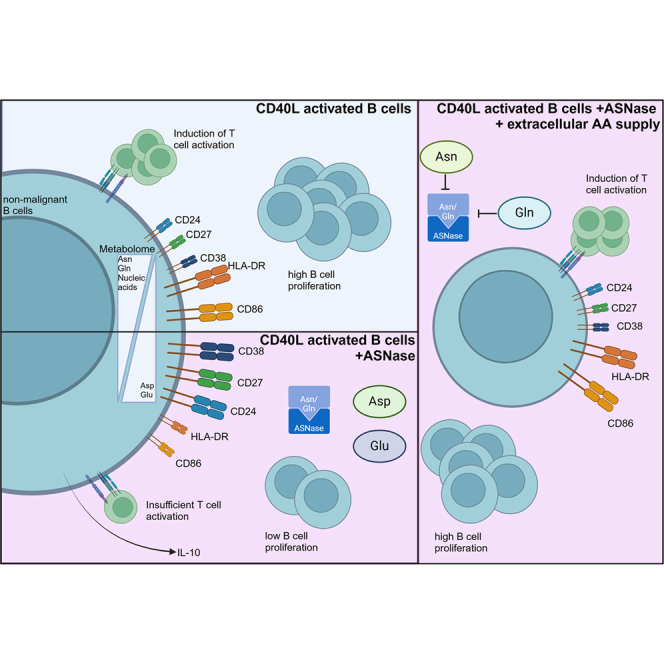

## Teaser

Biological sciences; Immunology; Immune response

## Introduction

Cellular metabolism plays a critical role for the differentiation and function of immune cells. While metabolic reprogramming is a well-established hallmark in activated T cells and macrophage subtypes, corresponding effects have also been recently observed in B cells.^1^ Following antigen binding and T cell mediated co-stimulation via the CD40 pathway, activated B cells proliferate and differentiate into antigen-presenting cells (APCs) and plasma cells. These changes are accompanied by profound metabolic changes toward increased production of energy equivalents, metabolites, and building blocks necessary for the synthesis of nucleic acids, proteins or other macromolecules.^1^^,^^2^ While proinflammatory responses are mediated by activated B cells that differentiated into APCs or memory B cell subsets, also anti-inflammatory, regulatory B cell subpopulations (Bregs) can develop from the B cell pool. Unlike regulatory T cells, Bregs are not governed by a single master transcription factor but are instead defined by their active capacity to suppress immune responses, e.g., by the secretion of immunosuppressive cytokines such as interleukin (IL)-10 or tumor growth factor beta (TGF-β).^3^ In humans, the capacity for IL-10 secretion is not restricted to a single B cell developmental stage, leading to the identification of multiple Breg phenotypes utilizing the surface markers CD24, CD27, and CD38. CD24 and CD38 are typically highly expressed on immature transitional B cells, while CD27 is a classic marker characterizing memory B cell populations. In this line, CD24^+^CD27^+^ and CD24^+^CD38^+^ B cells represent two phenotypes of Bregs with immunosuppressive capacities.^4^ On a metabolic level, Bregs exhibit lower glycolysis and oxidative phosphorylation rates compared to conventional activated B cells but rely on higher fatty acid oxidation, a feature that has also been linked to the immunoregulatory function of Tregs.^5^

Malignant B cells run similar metabolic programs with a high turnover of amino acids including glutamine and asparagine. While non-malignant B cells generate asparagine from glutamine in an ATP-dependent manner by asparagine synthetase (ASNS), malignant B cell fails to do so. In lymphoblastic B cell leukemia/lymphoma (B-ALL), asparagine becomes a conditionally essential amino acid due to defective ASNS activity of the blasts.^6^ Therefore, extracellular depletion of asparagine by L-asparaginase (ASNase) has been incorporated into multi-agent B-ALL treatment regimens for decades.^7^^,^^8^ For this purpose, multiple ASNase formulations have been developed and approved comprising the native *Escherichia coli* derived form, as well as polyethylene glycolated (PEG) ASNase with reduced immunogenicity and improved pharmacokinetic features.^6^^,^^9^^,^^10^^,^^11^ Additionally, ASNase has been shown to not only hydrolyze asparagine but also exerts glutaminolytic activity.^12^

Although the effects of ASNase on malignant B cells are well-documented, the impact on non-malignant B cells is still under investigation. While a very recent study has demonstrated ASNS upregulation and dependence on asparagine metabolism in murine germinal center B cells,^13^ most of the published data on ASNase effects on B cells date back 60 years or do not address human primary cells. In those early studies, ASNase treatment led to improved allograft skin transplant survival accompanied by reduced lymphocyte migration and proliferation in murine models.^14^^,^^15^ Furthermore, diminished humoral immune responses were observed in response to sheep red blood cells in mice.^16^

In the light of the crucial role of B cells for the induction of T cell-mediated immune responses, we here investigated the effects of ASNase on non-malignant, primary human B cells regarding their metabolic activity, metabolite profile and immunologic function as APCs. In an *in vitro* model of T cell dependent (CD40 mediated) B cell activation, we demonstrate that ASNase significantly decreased metabolic overall activities of CD40-B cells in a dose-dependent manner, which translated into reduced activation, expansion and APC-function of these cells In addition, we also observed the formation of Bregs in a proportion of the CD40-B cells. The immunosuppressive effects were present already at low ASNase concentrations corresponding to sub-therapeutic serum levels clinically used for the treatment of B-ALL and could be reversed by exogeneous supplementation of asparagine and glutamine to a slightly higher extend.

## Results

### ASNase inhibits proliferation and homotypic cluster formation of CD40-B cells in a dose-dependent manner

**Figure 1:**
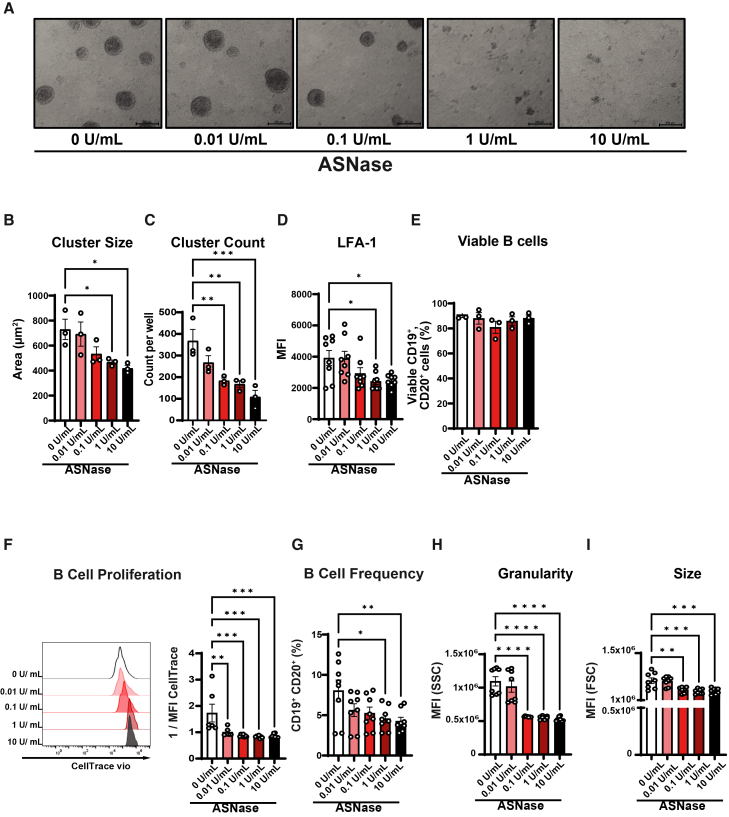
ASNase impairs CD40-B cell cluster formation and proliferation PBMCs from healthy blood donors were cultivated for 5 days and activated with CD40L multimers. Cultivation included ASNase treatment with different concentrations (0.01 U/mL–10 U/mL). (A) Representative microscopic image of cluster formation, scale represents 200 μm. (B) Cluster sizes and C cluster counts were analyzed using CellProfiler. (D) LFA-1 expression on CD19^+^ CD20^+^ B cells on day 5 of PBMC culture. (E) Viable CD19^+^ CD20^+^ B cells in PBMCs cell culture. (F) Representative histogram of CellTrace violet intensity and inverted MFIs showing B cell proliferation. (G) B cell frequency in PBMC culture on day 5 depending on ASNase concentration. (H) Granularity and (I) size were observed by flow cytometric analysis bar graphs show the respective MFIs. All experiments were performed in biological triplicates or quadruplicates, and biological replicates were not pooled. Data are represented as mean ± SEM. Statistical significance was calculated with one-way ANOVA (ref. 0 U/mL). Not significant (ns) *p* > 0.05; ∗*p* < 0.05; ∗∗*p* < 0.01; ∗∗∗*p* < 0.001; ∗∗∗∗*p* ≤ 0.0001.

To get insights into the overall effects of ASNase on CD40-B cells, we performed activation and proliferation experiments with PBMC-derived, CD40-B cells. To generate CD40-B cells, PBMCs were cultured for 5 days in IMDM including cyclosporine A, and insulin. ASNase ranging from 0 to 10 U/mL was added to the cultures from the beginning. At the end of the 5-day culture, homotypic CD40-B cell cluster formation was visibly reduced by ASNase treatment (Figure 1A). Quantification of cluster numbers and sizes confirmed a significant reduction of both parameters in a ASNase dose-dependent manner. Remarkably, this effect was already observed at the lowest ASNase concentration used (0.1 U/mL) and was further enhanced with increasing ASNase concentrations compared to the controls without ASNase (Figures 1B and 1C). To evaluate a potential mechanism for reduced homotypic cluster formation, we analyzed the expression of the lymphocyte function-associated antigen 1 (LFA-1) on CD40-B cells by flow cytometry. Here, we found LFA-1 expression significantly reduced with increasing ASNase concentrations (Figure 1D, gating strategy Figure S1). As reduced CD40-B cell cluster formation might be rooted in lower B cell numbers, we also analyzed B cell proliferation and viability under ASNase incubation during CD40-stimulation. While B cell viability was not affected by ASNase (Figure 1E), we observed a significant reduction of B cell proliferation activity (Figure 1F) resulting in lower numbers at the end of the stimulation experiment (Figure 1G). Finally, cellular granularity and size were determined by flow cytometry as morphological surrogates of the activation status and metabolic activity. Both were found reduced in ASNase treated CD40-B cells (Figures 1H and 1I). Collectively, ASNase reduces CD40-B cell numbers and cluster formation via lower proliferation activities and reduced LFA-expression but not via cell death induction.

### ASNase impairs antigen-presenting phenotype and function of CD40-B cells

**Figure 2:**
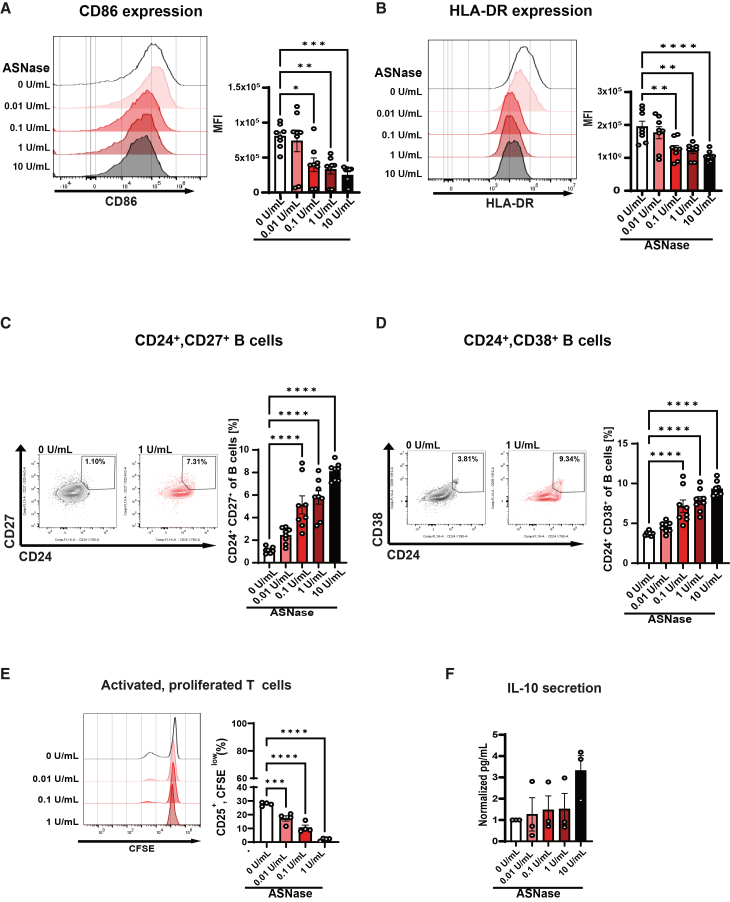
ASNase treatment hinders CD40-B cell activation inducing regulatory function thus impairing APC function PBMCs from healthy donors were cultivated for 5 days, activated with CD40L multimers and treated with ASNase at concentrations ranging from 0.01 U/mL to 10 U/mL. (A) Representative histogram of CD86 expression on CD19^+^ CD20^+^ B cells and quantitative analysis of CD86 MFI. (B) Representative histogram of HLA-DR expression on CD19^+^ CD20^+^ B cells and quantitative analysis of HLA-DR MFI. (C) Representative plots of CD24^+^ and CD27^+^ expression on CD19^+^ CD20^+^ B cells. Quantitative analysis of CD24^+^ and CD27^+^ Breg frequency. (D) Representative plots of CD24^+^ and CD38^+^ expression on CD19^+^ CD20^+^ B cells. Quantitative analysis of CD24^+^ and CD38^+^ Breg frequency. (E) Representative CFSE histogram analysis of T cells co-cultivated with ASNase pre-treated B cells and quantification of activated, proliferated T cells. (F) Normalized secretion of IL-10 into cell culture supernatant after days of CD40L activated B cell culture analyzed through ELISA. All experiments were performed in biological triplicates or quadruplicates, and biological replicates were not pooled. Data are represented as mean ± SEM. Statistical significance was calculated with one-way ANOVA (ref. 0 U/mL). Not significant (ns) *p* > 0.05; ∗*p* < 0.05; ∗∗*p* < 0.01; ∗∗∗*p* < 0.001; ∗∗∗∗*p* ≤ 0.0001.

To further assess the impact of ASNase on the biology and function of CD40-B cells, we measured the expression of classical co-stimulatory markers, i.e., CD86 and HLA-DR, on B cells after five days of incubation with CD40L-multimers and in different ASNase concentrations. Here, we found significantly reduced CD86 and HLA-DR expressions already at low ASNase concentrations (0.1 U/mL) which were further reduced in higher ASNase concentrations (Figures 2A and 2B). Additional analyses of CD21 and CD80 surface expression showed similar patterns of ASNase-mediated expression reduction (Figures S2A and S2B). For multidimensional flow data analyses of all ASNase concentrations and controls, sample data were concatenated and analyzed for CD86, HLA-DR, CD21, and CD80. Here, tSNE dimensionality reduction plots showed ASNase dose-dependent cluster formations with a gradient from 0 U/mL to 10 U/mL ASNase. Representative overlays of single marker expression profiles showed decreased expression of CD86, HLA-DR, and CD21 in all ASNase-treated samples (Figure S2C).

To investigate ASNase-mediated effects in other B cell subtypes, especially regulatory B cells, we also investigated CD24, CD38, and CD27 surface expression as well as the production of IL-10 and TGF-β as hallmarks of previously described Breg populations. Associated with rising ASNase concentrations we found remarkably increased numbers of CD24^+^CD27^+^ subsets (Figure 2C), as well as CD24^+^CD38^+^ Bregs (Figure 2D) at all ASNase concentrations and reaching statistically significance starting at 0.1 U/mL ASNase. Additionally, intracellular TGF-β expression and secreted IL-10 levels in supernatants were analyzed as a further markers of regulatory B cell function. Corresponding with Breg immunophenotypes, IL-10 amounts in the culture supernatants (Figure 2F) as well as intracellular TGF-β levels (Figure S2D) both tended to increase with rising ASNase concentrations. To correlate TGF-β expression to a certain Breg phenotype, we performed multiparameter analyses. Here, representative tSNE analysis suggested preferred clustering TGF-β expression in CD27 positive CD40-B cells under ASNase treatment (Figure S2E).

Based on these observations, we investigated the effect of ASNase on the ability to present antigens by pre-incubating B cells with ASNase for five days, followed by irradiating and co-culturing with donor-matched T cells (Figure 2E). The activation of T cells was measured by the fraction of CD25^+^ CFSE^−^ T cells. Here, pre-treatment of CD40-B cells with ASNase led to reduced T cell activation and proliferation already at 0.01 U/mL ASNase pre-treated CD40-B cells. Together, these results show that ASNase reduces CD40-B cell activation and differentiation into APCs and increases the formation of B cell subsets with a regulatory phenotype.

### ASNase induces metabolic restriction and quiescence in CD40-B cells

**Figure 3:**
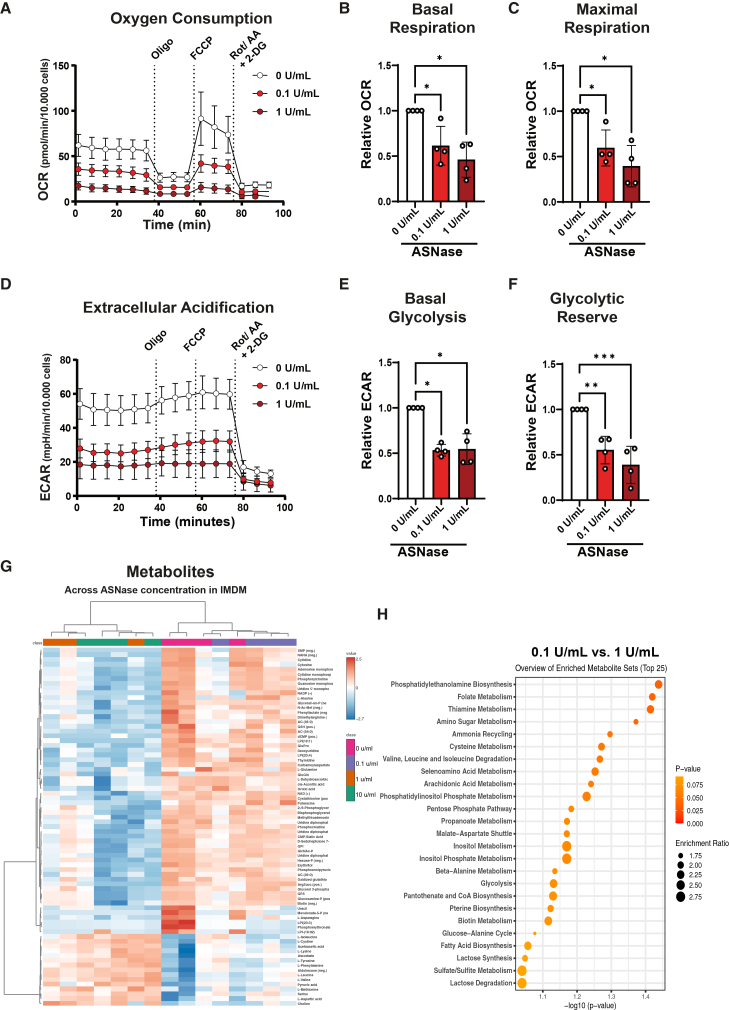
ASNase lowers metabolic overall activity and changes metabolic programs in CD40-B cells B cells were magnetically isolated from healthy donor PBMCs and activated by CD40-stimulation over 5 days. Metabolic activity of CD40-B cells was subsequently analyzed using extracellular flux analysis after 18 h ASNase treatment. (A) Representative oxygen consumption rates of CD40-B cells following sequential oligomycin, FCCP, and rotenone + antimycin a + 2-DG treatment. (B) Quantification of basal and (C) maximal respiration. (D) Representative extracellular acidification rates of B cells following sequential oligomycin, FCCP, and rotenone + antimycin a + 2-DG treatment. (E) Quantification of glycolysis and (F) glycolytic reserve. (G) Unsupervised clustering of metabolomics samples. (H) Enriched metabolite sets (0.1 U/mL vs. 1 U/mL). All experiments were performed in biological triplicates or quadruplicates, and biological replicates were not pooled. Data are represented as mean ± SEM. Statistical significance was calculated with one-way ANOVA (ref. 0 U/mL). Not significant (ns) *p* > 0.05; ∗*p* < 0.05; ∗∗*p* < 0.01; ∗∗∗*p* < 0.001; ∗∗∗∗*p* ≤ 0.0001.

To elucidate potential underlying mechanisms of ASNase-mediated suppressive effects on CD40-B cells we performed metabolic characterization of these cells based on oxygen consumption and extracellular acidification rates by extracellular flux analyses (Seahorse) as surrogates of mitochondrial activity and glycolysis, respectively. Of note, untreated CD40-B cells showed the highest oxygen consumption, while treatment with 0.1 U/mL and 1 U/mL ASNase gradually decreased oxygen consumption (Figure 3A). These differences were observed for both, the basal respiration (i.e., before the addition of FCCP) and the maximal respiration capacity (i.e., after addition of FCCP). Here, 1 U/mL ASNase nearly halved oxygen consumption rates (Figures 3B and 3C) and similar results were observed for the glycolytic activity (Figure 3D). To note, basal glycolytic activity as well as the glycolytic reserve was impaired beginning with ASNase concentrations of 0.1 U/mL (Figures 3E and 3F). Moreover, we observed that the addition of oligomycin or FCCP did not alter ECAR in any of the conditions suggesting that CD40-stimulation brought B cells to their maximal glycolytic capacity already. Glycolysis rates and glycolytic reserve capacities declined when cells were incubated with 0.1 U/mL or 1 U/mL ASNase. Oligomycin-mediated ATPase inhibition and FCCP induced uncoupling of the electron transport chain led only to little changes in ECAR in CD40-B cells, most likely as they were already at their maximum capacity glycolysis activities.

Based on these findings we sought to further elucidate alterations of the cellular metabolites mediated by ASNase treatment. Therefore, we performed LC-MS metabolomics from CD40-B cells after 5 days of CD40L stimulation and simultaneous ASNase incubation. Unsupervised clustering analyses on up and downregulated metabolites identified two major clusters composed of untreated and low-dose (0.1 U/mL) treated CD40-B cells forming one cluster and high concentrations (1 and 10 U/mL) forming the second large cluster (Figure 3G). Interestingly, the metabolite composition in low dose ASNase (0.1 U/mL) treated CD40-B cells was nearly identical to untreated controls except a distinct pattern of six metabolites. Of these, L-asparagine was strongly decreased already in 0.1 U/mL ASNase treated CD40-B cells confirming effective hydrolysis even at low dose ASNase levels. The other five suppressed metabolites in this pattern were uracil, mevalonate-5-P, LPI-(20:3), LPI-(18:02), and phosphoerythronate. This six-metabolite cluster was also observed to be regulated in high dose ASNase concentrations (1 and 10 U/mL) (Figure 3G). While ASNase treated CD40-B cells contained significantly less L-asparagine, L-aspartic acid as a cleavage product was found significantly higher (Figures S3A and S3B). Similarly, the cellular levels of L-glutamine declined under ASNase treatment in a dose-dependent manner, whereas glutamic acid contents remained unchanged (Figures S3C and S3D). Multivariate dimensionality reduction performed through partial least-squares discriminant analysis and based on all measured metabolites revealed distinct, dose-dependent formation of clusters and demonstrated similarities of low dose (0 and 0.1 U/mL) versus high dose (1 and 10 U/mL) ASNase treated cells (Figure S3E). Venn-diagram analyses of shared and private metabolite changes in each of the ASNase conditions and the control revealed six metabolites being shared in all ASNase treated samples but not the control. These metabolites were: aldohexose, serine, methionine, aspartic acid, leucine, and valine (Figure S3F). In ASNase treated cells the amino acids leucine, phenylalanine, valine, and aspartic acid as a product of ASNase treatment, were enriched. To further annotate metabolomic changes to functional pathways, we performed ontology analyses. Comparing 0.1 U/mL-treated CD40-B cells with 1 U/mL ASNase treated B cells revealed enrichment of pathways predominantly associated with proliferation and mitochondrial metabolism (Figure 3H). Based on this separation of non-treated and low dose treated CD40-B cells in contrast to probes treated with higher concentrations of ASNase we looked at enriched metabolite sets in CD40-B cells treated with 0.1 U/mL compared to 1 U/mL. Most enriched pathways were associated with proliferation (folate metabolism, purine biosynthesis, glycolysis and pentose phosphate pathway) or are linked with activation (phophatidylinositol signaling, inositol metabolism, arachidonic acid metabolism, amino sugar metabolism, and cysteine metabolism). Taken together, these findings indicate that ASNase, already at low concentrations, profoundly reduce the overall metabolic activity of CD40-B cells. Predominantly, metabolic pathways crucial for cellular proliferation and B cell activation were suppressed providing a rational for the reduced immunological function of stimulated B cells.

### Exogenous supply of asparagine and/or glutamine rescues ASNase-induced alterations in a dose-dependent manner

**Figure 4:**
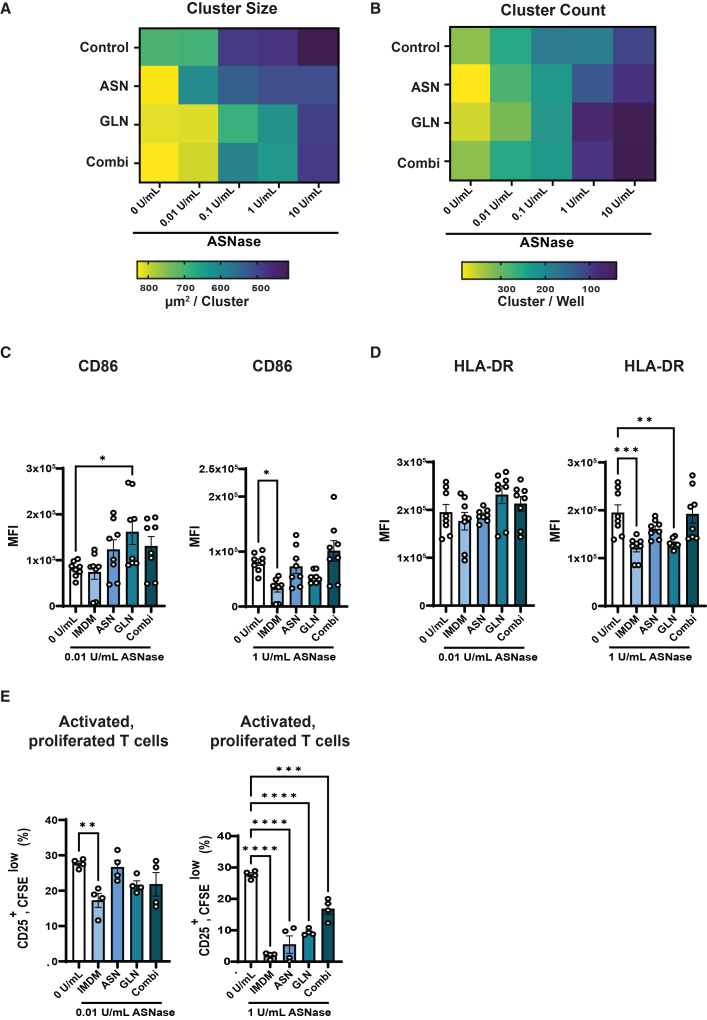
Exogenous supply of asparagine and/or glutamine rescues CD40-B cell cluster formation, activation and APC function Enriched B cells from healthy donors were stimulated with CD40L multimers over 5 days and in parallel treated with ASNase at concentrations varying from 0.01 U/mL to 10 U/mL. (A) Quantitative analysis of cluster sizes and (B) cluster counts in (non)-supplemented medium with different ASNase concentrations. (C) Quantitative analysis of CD86 MFI in (non)-supplemented medium with 0.01 U/mL or 1 U/mL ASNase. (D) Quantitative analysis of HLA-DR MFI in (non)-supplemented medium with 0.01 U/mL or 1 U/mL ASNase. (E) Quantification of activated and proliferated T cells co-cultivated with ASNase pre-treated B cells. All experiments were performed in biological triplicates or quadruplicates, and biological replicates were not pooled. Data are represented as mean ± SEM. Statistical significance was calculated with one-way ANOVA (ref. 0 U/mL). Not significant (ns) *p* > 0.05; ∗*p* < 0.05; ∗∗*p* < 0.01; ∗∗∗*p* < 0.001; ∗∗∗∗*p* ≤ 0.0001.

Given the strong shifts in proliferation, metabolic activities and APC function we asked if these effects are reversible through asparagine but also glutamine supply, since ASNase has been shown to hydrolyze both asparagine and glutamine. Similarly, to the previous experimental setting, CD40-B cells were generated and treated with ASNase but additionally supplemented with an excess of either asparagine or glutamine or a combination of both. Here, we found that the formation of homotypic CD40-B cell clusters could be rescued by both asparagine and glutamine supply (Figure S4A). However, quantification of cluster sizes and numbers revealed a distinct pattern of rescuing effects. Clusters were larger upon glutamine supplementation, with or without additional asparagine, and this effect could be observed in ASNase concentrations up to 1 U/mL (Figure 4A). In contrast, cluster counts were rescued from ASNase suppression up to 0.1 U/mL by both amino acids to a similar extend (Figure 4B). From these rescue experiments, we also analyzed the cell size and granularity of single CD40-B cells by flow cytometry, and we found a similar rescue pattern as observed before in the analyses of homotypic clusters (Figures S4B and S4C).

Next, we asked whether also the immunophenotype and APC function could be rescued by asparagine and/or glutamine supplementation. Based on our previous findings which showed that ASNase concentrations of 0.1 U/mL and 1 U/mL separated CD40-B cell-derived metabolites into two large clusters, we addressed this question in these two ASNase conditions. We observed that the expression of CD86 was significantly upregulated in low-dose ASNase (0.1 U/mL) treated CD40-B cells when glutamine, and to a lesser extent, also asparagine was supplemented. These effects were less prominent in 1 U/mL ASNase conditions (Figure 4C). In contrast, HLA-DR expression was almost unchanged by amino acid supplementation in both ASNase conditions (Figure 4D). When we screened for the frequency of Breg subsets, we saw that only glutamine, or a combination of asparagine and glutamine showed a decrease of this population (Figure S4D). To evaluate potential functional consequences on APC function, we performed mixed lymphocyte reaction assays (MLRs) with allogeneic isolated primary T cells. Here, the APC function was rescued in 1 U/mL ASNase treated CD40-B cells with stronger and additive effects of glutamine supplementation (Figure 4E). Overall, our data suggest, that exogenous supply of asparagine, glutamine, or their combination can partially rescue ASNase-mediated effects on CD40-B cells regarding activation, cluster formation and APC function with a tendency of glutamine being more effective.

### ASNase treatment mediates distinct metabolomic patterns in CD40-B cells

**Figure 5:**
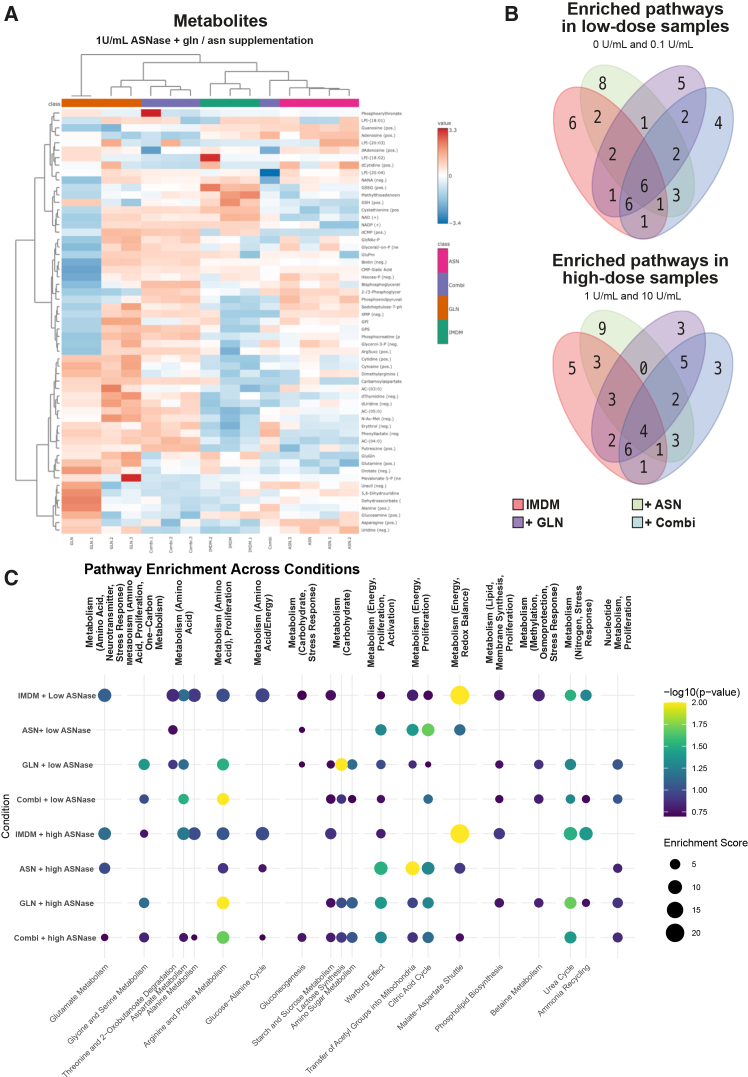
Supplementation of asparagine and/or glutamine readjust the metabolic profiles of CD40-B cells Magnetically isolated B cells from healthy donors were cultivated for 5 and activated with CD40L multimers. Cultivation included ASNase treatment with different concentrations (from 0.01 U/mL up to 10 U/mL). (A) Unsupervised clustering of metabolomics samples treated with 1 U/mL ASNase. (B) Venn-diagrams of enriched metabolomics pathways in samples treated with low dose (0 and 0.1 U/mL) and high dose (1 and 10 U/mL) ASNase. (C) Dot-plot depicting enriched pathways across conditions dot size depicting enrichment score and color depicting *p* value. Data are represented as mean ± SEM. Statistical significance was calculated with one-way ANOVA (ref. 0 U/mL). Not significant (ns) *p* > 0.05; ∗*p* < 0.05; ∗∗*p* < 0.01; ∗∗∗*p* < 0.001; ∗∗∗∗*p* ≤ 0.0001.

To better understand the amino acid supplementation effects on revived CD40-B cell cluster formation, APC phenotype and functionality, we finally analyzed changes in cellular metabolite patterns. The addition of asparagine alone or in combination with glutamine proved effective to bring up cellular asparagine and aspartic acid levels. Similarly, the addition of glutamine elevated cellular glutamine and glutamic acid levels (Figure S5A). Unsupervised cluster analysis of metabolites found in CD40-B cells treated with 1 U/mL ASNase and the respective supplementation groups and untreated control cells revealed a clear separation of clusters depending on the ASNase treatment and the amino acid supplementation. Moreover, metabolite patterns derived from glutamine supplementation only or in combination with asparagine showed high similarities (Figure 5A). Corresponding PLS-DA analysis showed that the previously seen metabolite cluster formation was only slightly affected by asparagine supplementation (Figure S3E), while glutamine containing supplementation changed metabolite clusters more profoundly (Figure S5B). Quantification of significantly over-represented metabolites from all experimental groups showed a separation low dose (0–0.1 U/mL) ASNase conditions from samples treated with higher doses (1–10 U/mL) (Figure 5A). Ontology analysis conducted with these sets of metabolites showed that samples treated with low dose ASNase enriched for pathways associated with proliferation, while samples treated with higher doses of ASNase affected metabolite recycling pathways, most likely to compensate for metabolic restriction (Figure S5C). Based on this metabolite clustering pattern (low dose versus high dose ASNase treatment), we screened these two groups for enriched pathways in asparagine and glutamine supplementation experiments. Here, Venn-diagram analysis resulted in an overlapping pattern of shared versus private metabolites in low and high dose ASNase conditions (Figure 5B). Detailed metabolic pathway enrichment analyses of all conditions showed that glutamate metabolism pathways were more prominently affected in ASNase high dose samples. In low dose ASNase treated samples, amino acid driven proliferation and activation pathways were affected to a greater extend compared to high dose samples. Coherently, membrane lipid biosynthesis was also more enriched in ASNase low dose samples, whereas nucleotide metabolism depended more on amino acid supplementation. Stress response associated pathways tended to be more prominent in ASNase high dose samples, whereas carbohydrate, lipid and nitrogen metabolism pathways were not found to be enriched in asparagine supplemented samples (Figure 5C). In summary, we observed distinct metabolic patterns depending on asparagine and glutamine supplementation and the dose of ASNase with pronounced impact on cell proliferation pathways in low dose ASNase-treated CD40-B cells and stress response pathways in high dose ASNase conditions.

## Discussion

In this study we investigated the effects of ASNase as a key agent in B-ALL treatment on non-malignant primary human B cells. We found that ASNase induced metabolic restriction impairs CD40-B cell activation, proliferation and APC function *in vitro*. Also, we observed that part of the ASNase treated stimulated B cells shifted toward a regulatory immune phenotype and expressed the immunosuppressive cytokines IL-10 and TGF-β. Of note, these effects were detectable at concentrations below therapeutic serum levels targeted in B-ALL treatment and proved reversible when glutamine and/or asparagine were supplemented. Thus, this study extends previous pre-clinical data on ASNase-mediated immunomodulation.^14^^,^^15^^,^^16^

Non-malignant B cells possess functional asparagine synthetase (ASNS) activity and are therefore not reliant on extracellular asparagine supply. This might explain, why ASNase treated non-malignant CD40-B cells showed lower proliferation capacities but did not display increased cell death rates in our experiments. However, B cells undergo metabolic reprogramming following their activation,^17^ and amino acid uptake and metabolism are in the core of these processes^18^ to provide cellular mass and biomolecule generation.^19^^,^^20^ This is in line with current knowledge on immunometabolism in T cells, which has been investigated to more extend compared to B cell metabolism. In T cells, the consequences of asparagine restriction depend obviously on the specific context, as recent studies have reported partly conflicting outcomes, i.e., T cell activation or functional inhibition.^21^^,^^22^^,^^23^ In non-malignant B cells, the effects of asparagine depletion or restriction have only recently been analyzed.^20^ Here, Yazicioglu et al. found asparagine metabolism essential for B cell differentiation and maturation during the germinal center (GC) reaction in a preclinical mouse model. Both, suppression of cellular asparagine synthesis by a B cell-specific ASNS-gene knock out or via environmental depletion of asparagine, prevented metabolic re-programing of murine B cells and formation of proper post-GC B cells.^13^ In line with this, our study extends the analyses to human primary B cells and to a model of T cell-mediated B cell activation.

In addition to activated B cells, classic APCs such as dendritic cells and macrophages also rely on amino acid metabolism to fully exert APC function. Imbalanced blood plasma amino acid levels disturb fatty acid metabolism in dendritic cells causing delayed maturation and impaired APC function,^24^ and another study showed that ASNase disturbs phagocytosis, proliferation, and antigen presentation in macrophages.^25^ Our findings in CD40-B cells, coincide with these observations made in classical APCs. However, a direct mechanistic link of our findings derived from Seahorse life-cell analyses and metabolomics cannot be drawn given the complexity of potential mechanisms involved. A plausible model might be that ASNase-mediated asparagine and preparation-dependent glutamine depletion, limits the amino acid availability required for CD40-driven B cell activation.^26^ Activated CD40-B cells depend on increased glucose and glutamine uptake, oxidative metabolism, glycolysis, mTORC1 activity, and MYC-associated anabolic programs to support effector differentiation.^27^ In parallel, amino acid limitation can activate GCN2/eIF2α-ATF4 stress signaling and attenuate global translation, while asparagine itself has been shown to regulate amino acid exchange, mTORC1 activation, nucleotide synthesis, and protein synthesis.^28^ These pathways might converge and could provide a plausible mechanistic bridge between ASNase-induced metabolic restriction and the reduced antigen-presenting capacity and function described in this study. In addition the differential sensitivity of CD86 and HLA-DR seen in our experiments may reflect their distinct regulatory architecture. HLA-DR expression depends on CIITA-driven MHC-II transcription and on coordinated synthesis, assembly, trafficking, and surface maintenance of the MHC-II pathway, whereas CD86 is regulated through partially distinct activation-dependent and post-translational mechanisms.^29^ Both CD86 and MHC-II are substrates of MARCH-I-dependent ubiquitination and lysosomal trafficking in professional APCs, but their transcriptional regulation and protein-biogenesis requirements differ.^30^ Therefore, ASNase may affect HLA-DR and CD86 through overlapping but non-identical mechanisms, potentially explaining their divergent sensitivity to ASNase and amino acid supplementation.

An often overseen but important effect of ASNase activity is hydrolyzation of glutamine which has been shown critical for its therapeutic activity in B-ALL,^12^ but also impairs non-malignant B cell function.^31^ We observed glutamine supplementation to be a major, and seemingly superior factor to compensate for ASNase-mediated inhibition of CD40-B cell proliferation and APC function. Along with this finding, other studies have observed that asparagine deficiency cannot be fully compensated in glutamine depleted conditions making asparagine conditionally essential.^32^ In addition to serving as a nitrogen donor for intracellular asparagine synthesis by asparagine synthetase in ASNS-competent cells, glutamine also replenishes tricarboxylic acid cycle intermediates, supports nucleotide production, and restores glutathione-dependent redox balance.^32^^,^^33^^,^^34^ Therefore, reversibility of ASNase effects in non-malignant CD40-B cells can be prominently mediated also by glutamine and functions without asparagine supplementation possibly due to the functional ASNS activity in these cells which catalyzes asparagine synthesis from aspartate and glutamine.^6^ The relative importance of these mechanisms will depend on cellular ASNS expression, transporter activity, and the glutaminase component of the L-ASNase preparation used, and should be considered when interpreting the rescue experiments in our study. While ASNase is a cornerstone of B-ALL treatment, it has not reached clinical approval for the treatment of other cancer entities studied (ovarian and pancreatic cancer) due to low efficacy and/or the disadvantageous side effect profile,^35^ e.g., hepatotoxicity and coagulation disturbances, associated with the conventional ASNase doses. However, we identified four ongoing studies (clinicaltrials.gov: NCT01523808; NCT02195180; NCT03665441; NCT05034627) that investigate the role of ASNase in combinatorial treatments against solid (pancreatic) cancer. In this study, we used a range of ASNase concentrations that cover a range of concentrations below and above the clinical steady state target level (>0.1 U/mL). Here, we found that 10-fold lower ASNase concentrations effectively suppressed the proliferation and APC function of CD40-B cells. In addition, we found that low dose ASNase treatment is associated with the formation of B cell subpopulations with a Breg phenotype, which might add to the immunosuppressive effects of ASNase. Therefore, we hypothesize that low dose ASNase might be a therapeutic option for B cell dependent severe autoimmune diseases such as lupus erythematosus, chronic graft-versus-host disease, or bullous skin diseases.^36^^,^^37^^,^^38^ To our knowledge, ASNase has not been clinically evaluated as an immunosuppressant so far, and no ongoing studies are registered to this topic at www.clinicaltrials.gov. On the other hand, and pointing into that direction, a recently preprinted study reported on ASNase treatment in a murine model of multiple sclerosis.^39^ Here, the authors found that depletion of extracellular asparagine by ASNase suppressed self-reactive T cells and ameliorated autoimmunity in multiple sclerosis affected mice. Similar effects were shown by Hope et al. in another preclinical study.^40^ Given its fundamentally different mode of action, ASNase—especially in lower doses than used in cancer treatment—might therefore enrich the immunosuppressant armamentarium if proven safe and effective in clinical trials. Where corticosteroids, calcineurin inhibitors, antimetabolites, and biologics act primarily through signaling-pathway inhibition or via interference with nucleotide synthesis, ASNase acts through metabolic restriction, depletion of extracellular asparagine and glutamine, and the integrated stress response (ISR). The fact that supplementation of asparagine or glutamine can ameliorate ASNase effects might also open the window of better therapeutic activity and side effect control.

### Limitations of the study

Limitations of this study are certainly the *in vitro* setting, limited cohort size, and that we cannot completely rule out possible additional effects of other nutrients or metabolites present in the media. However, in the light of the already published data generated from murine models that are principally in line with our observations render the latter possibility rather unlikely. Nevertheless, confirmatory preclinical studies in a mouse model of B cell-mediated autoimmunity, e.g., multiple sclerosis, are needed. Such studies are also warranted to evaluate potential toxicities of low dose ASNase treatment before proceeding to a clinical trial. Furthermore, an open question is whether the observed ASNase effects follow a linear dose dependency, or if a certain threshold exists that might be also exploited in advantage to separate immunomodulatory effects from toxicities.

Conclusively, we show profound metabolic and immunomodulatory effects mediated by ASNase in human, CD40-activated B cells. We speculate that the low doses needed to induce these effects might reduce the side effects seen in current B-ALL treatment usage, which might open the way to investigate ASNase as an immunomodulatory drug in future trials.

## Resource availability

### Lead contact

Requests for further information and resources should be directed to and will be fulfilled by the lead contact Prof. Dr. Sebastian Theurich, sebastian.theurich@med.uni-muenchen.de.

### Materials availability

This study did not generate new unique reagents.

### Data and code availability

• Data: The metabolomics data for this study are uploaded and accessible at Metabolomics Workbench: An international repository for metabolomics data and metadata, metabolite standards, protocols, tutorials and training, and analysis tools (2016)'. [PubMed: https://www.ncbi.nlm.nih.gov/pubmed/26467476/] This study is available at the NIH Common Fund’s National Metabolomics Data Repository (NMDR) Website, the Metabolomics Workbench, https://www.metabolomicsworkbench.org where it has been assigned Study ID ST004506. The data can be accessed directly via its Project DOI: https://doi.org/10.21228/M86S0P. This work is supported by NIH grant U2C-DK119886. The study is available for review at https://dev.metabolomicsworkbench.org:22222/data/DRCCMetadata.php?Mode=Study&StudyID=ST004506&Access=OjzL8334 Regards, NIH Common Fund’s National Metabolomics Data Repository (NMDR).
• Code: This paper does not report original code.
• All other: Any additional information required to reanalyze the data reported in this paper is available from the lead contact upon request.

## Acknowledgments

This work received a research grant by 10.13039/501100014841medac GmbH (Wedel, Germany), and we would like to dedicate this work to the memory of Dr. Monika Essink who strongly supported us with her kindness and curiosity. This work was also supported by 10.13039/501100001659German Research Foundation (10.13039/501100001659DFG), SFB-TRR338 -1 2021 (“Letsimmun” project no. 452881907). D.M.C.d.S. received the Walter Benjamin Fellowship from the 10.13039/501100001659Deutsche Forschungsgemeinschaft (DFG, German Research Foundation, no. 525171148). Further support was granted by the Thomas Kirch Stiftung, the Bruno and Helene Jöster Foundation (360° CART) and by the Maria Sklodowska Research Training Network of the European Union (T-OP, project no. 955575). We are grateful to Dr. Benjamin Tast, Core Facility Flow Cytometry (iFlow), 10.13039/501100005722LMU Biomedical Center, Munich, and Can Pinar (Cytolution, Tübingen) for excellent technical support. Gratitudes also to Lucia Guldner for critical reading the manuscript.

## Author contributions

Conceptualization, A.H., M.A.G.-M., and S.T.; experimental investigation, A.H., M.A.G.-M., P.B., N.H., K.F., D.M.C.d.S., M.F., A.T., J.T., A.C., L.V., and S.O.; data analysis and visualization, A.H., L.V., and M.K.; methodology, A.H., M.A.G.-M., and S.T.; manuscript writing and editing: A.H., D.M.C.d.S., S.T., H.S., M.V., and M.v.B.-B. All authors read and approved the final manuscript.

## Declaration of interests

The authors declare no competing interests.

## STAR★Methods

### Key resources table

**Table:** 

REAGENT or RESOURCE	SOURCE	IDENTIFIER
**Antibodies**		

Brilliant Violet 421 anti-human CD80	Biolegend	305222
Brilliant Violet 650 anti-human CD86	Biolegend	305427
PE/Cyanine7 anti-human CD19	Biolegend	363012
PE/Dazzle tm 594 anti-human CD20	Biolegend	302347
APC/Cyanine7 anti-human CD21	Biolegend	354927
Brilliant Violet 421 anti-human CD24	Biolegend	311121
Brilliant Violent 605 anti-human CD27	Biolegend	302829
Brilliant Violent 785 anti-human CD38	Biolegend	303529
PE anti-human IL-10	Biolegend	501403
FITC anti-human LAP (TGF-beta1)	Biolegend	300009

**Chemicals, peptides, and recombinant proteins**		

recombinant human IL-4	ImmunoTools	11340045

**Critical commercial assays**		

Pan-B Isolation Kit	Miltenyi Biotech	130-101-638
CD40-ligand multimers	Miltenyi Biotech	130-098-776
IL-10 ELISA MAX	Biolegend	430604
Zombie violet Fixable Viability Kit	Biolegend	423112
EasySep T-cell enrichment kit	StemCell Technologies	17951

**Deposited data**		

Metabolomics Data (Metabolomics Workbench)	Newly generated from CD40B cells	ID ST004506

**Software and algorithms**		

FlowJo	BD biosciences	N/A
MetaboAnalyst	MetaboAnalyst.ca	N/A
GraphPad Prism	GraphPad Prism	N/A

### Experimental model and study participant details

A total of 10 healthy blood donors (males *n* = 5; females *n* = 5) with a median age of 31 (range 20–60 years) were used for the experiments. Biological replicates were analyzed individually. The study was conducted in accordance with the Declaration of Helsinki and approved by the institutional review boards (11–116, University of Cologne and 21–0082 LMU Munich). From all donors informed consent was obtained prior to sampling.

### Method details

#### CD40-B cell cultures and treatments

To generate CD40-B cells for microscopy analyses, extracellular flux assays, and metabolomics, peripheral B cells were enriched from PBMCs by negative selection (Pan-B Isolation Kit, Cat-No. 130-101-638, Miltenyi Biotech, Germany), seeded at a density of 1 × 106 B cells/mL in 24 well plates and cultured over 5 days without cell splitting at 37°C, 5% CO2 humidified atmosphere in Iscove's modified Dulbecco medium (IMDM) (GIBCO by Life Technologies, California, United States) supplemented with 10% heat-inactivated human serum (pooled serum derived from the PBMC donors), penicillin/streptomycin (GIBCO by Life Technologies, California, USA), 50 U/mL recombinant human IL-4 (ImmunoTools, Cat-No 11340045, Friesoythe, Germany), 5 mg/mL insulin (Novo Nordisk Pharma GmbH, Cat-No 0417642, Mainz, Germany), and CD40-ligand multimers (Miltenyi Biotech, Cat-No. 130-098-776, Bergisch-Gladbach, Germany).

For flow cytometry analyses, CD40-B cells were directly generated from PBMCs by additional cyclosporine-A incubation at 0.63 μg/mL (Sigma-Aldrich, Missouri, United States). Here, PBMCs were seeded at a density of 3 × 106 cells/mL in 24 well plates and cultured over 5 days at the same conditions as performed in B cell selected cultures mentioned above.

For experiments with ASNase treatment (Medac, Hamburg, Germany), glutamine or asparagine supplementation (Sigma Aldrich, Germany) the respective compounds were added to the cell culture from the beginning. L-Asparaginase was used in concentrations from 0.01 U/mL to 10 U/mL. Glutamine or asparagine was supplemented at a final concentration of 2 mM respectively.

#### Microscopy and image analyses

The microscopic evaluation of homotypic cluster formation of CD40-B cells was performed after 5 days of cell culture using the Leica Thunder Imaging System (Leica Microsystems, Wetzlar, Germany). Whole wells were evaluated automatically using Leica LAS X software with 100× magnification. Evaluation of microscopy pictures was performed using Leica Thunder imaging software (Leica Microsystems, Life Science version) and CellProfiler (CellProfiler, version 4).

#### Flow cytometry

For flow cytometric analyses 2 × 105 cells per sample were washed twice (centrifugation 550 × g, 5 min) in Dulbecco's Phosphate Buffered Saline (DPBS) and stained with 2 μg/mL of each primary and fluorochrome-conjugated antibody in 20 μL DPBS per sample. Samples were incubated for 20 min at 4°C in the dark, washed and resuspended in fresh DPBS before flow cytometric analysis.

For proliferation assessment, cells were labeled with carboxyfluoresceinsuccinylimidyl (CFSE) or CellTrace violet (Thermo Fisher Scientific, Massachusetts, United States) according to the manufacturer's protocol.

Flow cytometry data were acquired with either the Gallios 10-colour flow cytometer (Beckman Coulter, California, United States) or the Cytoflex LX flow cytometer (Beckman Coulter, California, United States). Depending on the flow cytometer used, data were analyzed with the Kaluza software package (Beckmann Coulter, version 1.1), FlowJo (BD Biosciences, version 10) or Cytolution (Cytolytics, Tübingen, Germany) respectively.

#### Mixed lymphocyte reaction

Activated CD40-B cells were used as antigen-presenting cells after 5 days of CD40-stimulation and thorough washing at the end of the activation period. Allogeneic T cells were purified by negative immunomagnetic selection (EasySep T-cell enrichment kit, Catalog No: 17951, StemCell Technologies, Grenoble, France) from peripheral blood of healthy donors. A total of 2 × 104 irradiated (26 Gy) CD40-B cells were co-cultured with 10 × 104 allogeneic T cells in a final volume of 200 μL per well in 96-well round bottom plates. Prior to co-culture, T cells were labeled with CellTrace/CFSE as previously described. After 5 days, cells were harvested and subjected to flow cytometry for proliferation analysis and immuno-phenotyping.

#### Enzyme-linked immunosorbent assay (ELISA)

IL-10 secretion from CD40-B cells was evaluated using ELISA. Supernatants from cell cultures were taken on day 5 and analyzed using ELISA MAX (Biolegend, California, United States) according to manufacturer's protocol. Absorption was measured with the Cytation 1 (Biotek Instruments, Vermont, United States).

#### Metabolic extracellular flux assays (Seahorse)

Magnetically sorted CD40-B cells were treated with asparaginase for 18h and 1.5 × 105 cells were plated on a poly-D-lysine coated 96-well Seahorse utility plate (Agilent, California, United States). Surrogate parameters for glycolytic and respiratory activities, i.e., extracellular acidification rates (ECARs) and oxygen consumption rates (OCRs), were measured at base line and immediately after sequential addition of certain inhibitors: i) oligomycin, ii) carbonyl cyanide-*p*-trifluoromethoxyphenylhydrazone (FCCP), and iii) rotenone/antimycin A combined with 2-deoxyglucose, each with a final concentration of 1 μM. Measurements were conducted with the standard setting for xFE96 analyzers (Agilent). Each measurement cycle included calibration, mixing and measurements for ca. 3 min each, each interval takes approximately 20 min after injection.

#### Metabolomics, LC/MS analyses

Magnetically sorted CD40-B cells (1 × 106 cells) were washed twice with PBS and cell pellets were used for metabolomic analysis. Water-soluble metabolites were extracted from cell pellets with 500 μL ice-cold MeOH/H2O (80/20, v/v) containing 0.01 μM lamivudine and 1 μM each of D4-succinate, D5-glycine, D2-glucose and 15N-glutamate as external standards (Sigma-Aldrich, St. Louis, USA). After centrifugation of the resulting homogenates, supernatants were evaporated in a rotary evaporator (Savant, Thermo Fisher Scientific, Waltham, USA). Dry sample extracts were redissolved in 100 μL 5 mM NH4OAc in CH3CN/H2O (50/50, v/v). 15 μL supernatant was transferred to LC-vials. For LC-MS analysis 3 μL of each sample was applied to a XBridge Premier BEH Amide (2.5 μm particles, 100 × 2.1 mm) UPLC-column (Waters, Dublin, Ireland). Metabolites were separated with Solvent A, consisting of 5 mM NH4OAc in CH3CN/H2O (40/60, v/v) and solvent B consisting of 5 mM NH4OAc in CH3CN/H2O (95/5, v/v) at a flow rate of 200 μL/min at 45°C by LC using a DIONEX Ultimate 3000 UHPLC system (Thermo Fisher Scientific, Bremen, Germany). A linear gradient starting after 2 min with 100% solvent B decreasing to 0% solvent B within 23 min, followed by 17 min 0% solvent B and a linear increase to 100% solvent B in 1 min. Recalibration of the column was achieved by 7 min prerun with 100% solvent B before each injection. Ultrapure H2O was obtained from a Millipore water purification system (Milli-Q Merck Millipore, Darmstadt, Germany). HPLC-MS solvents, LC-MS NH4OAc, standards and reference compounds were purchased from Merck.

All MS-analyses were performed on a high-resolution mass spectrometer equipped with a HESI probe (Q Exactive, Thermo Fisher Scientific, Bremen, Germany) in alternating positive- and negative full MS mode with a scan range of 69.0–1000 m/z at 70K resolution and the following ESI source parameters: sheath gas: 30, auxiliary gas: 1, sweep gas: 0, aux gas heater temperature: 120°C, spray voltage: 3 kV, capillary temperature: 320°C, S-lens RF level: 50. XIC generation and signal quantitation was performed using TraceFinder V 5.1 (Thermo Fisher Scientific, Bremen, Germany) integrating peaks which corresponded to the calculated monoisotopic metabolite masses (MIM ± H+ ± 3 mMU).

### Quantification and statistical analysis

All experiments were conducted with at least three biological triplicates which were analyzed individually. Biological replicates were analyzed individually and not pooled. Statistical analysis was performed using GraphPad Prism (GraphPad, version 9.3). Three or more groups were analyzed by Kruskal-Wallis with Dunn's multiple comparison test. Three or more groups with more than one variable were analyzed using two-way ANOVA with Tukey’s multiple comparison test. *p* values ≤0.05 were considered statistically significant. Data are represented as mean ± SEM. Statistical significance was calculated with one-way ANOVA (ref. 0 U/mL). Not significant (ns) *p* > 0.05; ∗*p* < 0.05; ∗∗*p* < 0.01; ∗∗∗*p* < 0.001; ∗∗∗∗*p* ≤ 0.0001.

#### Additional resources

For analysis of metabolomics data MetaboAnalyst.ca (version 6.0) was used.

## References

[1] Boothby M., Rickert R.C. (2017). Metabolic Regulation of the Immune Humoral Response. Immunity.

[2] Zarnegar B., He J.Q., Oganesyan G., Hoffmann A., Baltimore D., Cheng G. (2004). Unique CD40-mediated biological program in B cell activation requires both type 1 and type 2 NF-κB activation pathways. Proc. Natl. Acad. Sci. USA.

[3] Catalán D., Mansilla M.A., Ferrier A., Soto L., Oleinika K., Aguillón J.C., Aravena O. (2021). Immunosuppressive Mechanisms of Regulatory B Cells. Front. Immunol..

[4] Hasan M.M., Thompson-Snipes L., Klintmalm G., Demetris A.J., O'Leary J., Oh S., Joo H. (2019). CD24hiCD38hi and CD24hiCD27+ Human Regulatory B Cells Display Common and Distinct Functional Characteristics. J. Immunol..

[5] Iperi C., Bordron A., Dueymes M., Pers J.O., Jamin C. (2021). Metabolic Program of Regulatory B Lymphocytes and Influence in the Control of Malignant and Autoimmune Situations. Front. Immunol..

[6] Lomelino C.L., Andring J.T., McKenna R., Kilberg M.S. (2017). Asparagine synthetase: Function, structure, and role in disease. J. Biol. Chem..

[7] Maese L., Rau R.E. (2022). Current Use of Asparaginase in Acute Lymphoblastic Leukemia/Lymphoblastic Lymphoma. Front. Pediatr..

[8] Sandley M., Angus J. (2023). Asparaginase therapy in patients with acute lymphoblastic leukemia: expert opinion on use and toxicity management. Leuk. Lymphoma.

[9] Fu C.H., Sakamoto K.M. (2007). PEG-asparaginase. Expert Opin. Pharmacother..

[10] Maese L., Loh M.L., Choi M.R., Lin T., Aoki E., Zanette M., Agarwal S., Iannone R., Silverman J.A., Silverman L.B. (2023). Recombinant Erwinia asparaginase (JZP458) in acute lymphoblastic leukemia: results from the phase 2/3 AALL1931 study. Blood.

[11] Métayer L.E., Brown R.D., Carlebur S., Burke G.A.A., Brown G.C. (2019). Mechanisms of cell death induced by arginase and asparaginase in precursor B-cell lymphoblasts. Apoptosis.

[12] Chan W.K., Horvath T.D., Tan L., Link T., Harutyunyan K.G., Pontikos M.A., Anishkin A., Du D., Martin L.A., Yin E. (2019). Glutaminase Activity of L-Asparaginase Contributes to Durable Preclinical Activity against Acute Lymphoblastic Leukemia. Mol. Cancer Therapeut..

[13] Yazicioglu Y.F., Marin E., Andrew H.F., Bentkowska K., Johnstone J.C., Mitchell R., Wong Z.Y., Zec K., Fergusson J., Borsa M. (2024). Asparagine availability controls germinal center B cell homeostasis. Sci. Immunol..

[14] Friedman H., Chakrabarty A.K. (1971). Immunosuppressive effects of bacterial asparaginase on antibody formation and allograft immunity. Immunology.

[15] Weksler M.E., Weksler B.B. (1971). Studies on the immunosuppressive properties of asparaginase. Immunology.

[16] Nelson S.D., Lee M.B., Bridges J.M. (1970). Immunosuppressive activity of L-asparaginase in mice. Transplantation.

[17] Patke A., Mecklenbräuker I., Erdjument-Bromage H., Tempst P., Tarakhovsky A. (2006). BAFF controls B cell metabolic fitness through a PKCβ- and Akt-dependent mechanism. J. Exp. Med..

[18] Kelly B., Pearce E.L. (2020). Amino assets: how amino acids support immunity. Cell Metab..

[19] Hosios A.M., Hecht V.C., Danai L.V., Johnson M.O., Rathmell J.C., Steinhauser M.L., Manalis S.R., Vander Heiden M.G. (2016). Amino Acids Rather than Glucose Account for the Majority of Cell Mass in Proliferating Mammalian Cells. Dev. Cell.

[20] Johnstone J.C., Yazicioglu Y.F., Clarke A.J. (2024). Fuelling B cells: dynamic regulation of B cell metabolism. Curr. Opin. Immunol..

[21] Gnanaprakasam J.N.R., Kushwaha B., Liu L., Chen X., Kang S., Wang T., Cassel T.A., Adams C.M., Higashi R.M., Scott D.A. (2023). Asparagine restriction enhances CD8+ T cell metabolic fitness and antitumoral functionality through an NRF2-dependent stress response. Nat. Metab..

[22] Hope H.C., Brownlie R.J., Fife C.M., Steele L., Lorger M., Salmond R.J. (2021). Coordination of asparagine uptake and asparagine synthetase expression modulates CD8+ T cell activation. JCI Insight.

[23] Ma S., Ming Y., Wu J., Cui G. (2024). Cellular metabolism regulates the differentiation and function of T-cell subsets. Cell. Mol. Immunol..

[24] Kakazu E., Kondo Y., Kogure T., Ninomiya M., Kimura O., Ueno Y., Shimosegawa T. (2013). Plasma amino acids imbalance in cirrhotic patients disturbs the tricarboxylic acid cycle of dendritic cell. Sci. Rep..

[25] Song P., Wang Z., Zhang X., Fan J., Li Y., Chen Q., Wang S., Liu P., Luan J., Ye L., Ju D. (2017). The role of autophagy in asparaginase-induced immune suppression of macrophages. Cell Death Dis..

[26] Egawa T., Bhattacharya D. (2019). Regulation of metabolic supply and demand during B cell activation and subsequent differentiation. Curr. Opin. Immunol..

[27] Krall A.S., Xu S., Graeber T.G., Braas D., Christofk H.R. (2016). Asparagine promotes cancer cell proliferation through use as an amino acid exchange factor. Nat. Commun..

[28] Ravindran R., Khan N., Nakaya H.I., Li S., Loebbermann J., Maddur M.S., Park Y., Jones D.P., Chappert P., Davoust J. (2014). Vaccine activation of the nutrient sensor GCN2 in dendritic cells enhances antigen presentation. Science.

[29] León Machado J.A., Steimle V. (2021). The MHC Class II Transactivator CIITA: Not (Quite) the Odd-One-Out Anymore among NLR Proteins. Int. J. Mol. Sci..

[30] Bandola-Simon J., Roche P.A. (2023). Regulation of MHC class II and CD86 expression by March-I in immunity and disease. Curr. Opin. Immunol..

[31] Le A., Lane A.N., Hamaker M., Bose S., Gouw A., Barbi J., Tsukamoto T., Rojas C.J., Slusher B.S., Zhang H. (2012). Glucose-Independent Glutamine Metabolism via TCA Cycling for Proliferation and Survival in B Cells. Cell Metab..

[32] Pavlova N.N., Hui S., Ghergurovich J.M., Fan J., Intlekofer A.M., White R.M., Rabinowitz J.D., Thompson C.B., Zhang J. (2018). As Extracellular Glutamine Levels Decline, Asparagine Becomes an Essential Amino Acid. Cell Metab..

[33] Hlozkova K., Vasylkivska M., Boufersaoui A., Marzullo B., Kolarik M., Alquezar-Artieda N., Shaikh M., Alaei N.F., Zaliova M., Zwyrtkova M. (2024). Rewired glutamate metabolism diminishes cytostatic action of L-asparaginase. Cancer Lett..

[34] Yoo H.C., Yu Y.C., Sung Y., Han J.M. (2020). Glutamine reliance in cell metabolism. Exp. Mol. Med..

[35] Van Trimpont M., Peeters E., De Visser Y., Schalk A.M., Mondelaers V., De Moerloose B., Lavie A., Lammens T., Goossens S., Van Vlierberghe P. (2022). Novel Insights on the Use of L-Asparaginase as an Efficient and Safe Anti-Cancer Therapy. Cancers (Basel).

[36] Zhu H.Q., Xu R.C., Chen Y.Y., Yuan H.J., Cao H., Zhao X.Q., Zheng J., Wang Y., Pan M. (2015). Impaired function of CD19(+) CD24(hi) CD38(hi) regulatory B cells in patients with pemphigus. Br. J. Dermatol..

[37] van der Vlugt L.E.P.M., Mlejnek E., Ozir-Fazalalikhan A., Janssen Bonas M., Dijksman T.R., Labuda L.A., Schot R., Guigas B., Möller G.M., Hiemstra P.S. (2014). CD24(hi)CD27(+) B cells from patients with allergic asthma have impaired regulatory activity in response to lipopolysaccharide. Clin. Exp. Allergy.

[38] Blair P.A., Noreña L.Y., Flores-Borja F., Rawlings D.J., Isenberg D.A., Ehrenstein M.R., Mauri C. (2010). CD19(+)CD24(hi)CD38(hi) B cells exhibit regulatory capacity in healthy individuals but are functionally impaired in systemic Lupus Erythematosus patients. Immunity.

[39] Georgiev P., Johnson S., Kurmi K., Hu S.H., Han S., Patterson D., Nguyen T.H., Huang L., Liang D., Goldman N. (2025). Depletion of extracellular asparagine impairs self-reactive T cells and ameliorates autoimmunity in a murine model of multiple sclerosis. bioRxiv.

[40] Aldoss I., Douer D. (2020). How I treat the toxicities of pegasparaginase in adults with acute lymphoblastic leukemia. Blood.

