# Supplementary material for: L-asparaginase reversibly suppresses CD40-mediated B cell activation and antigen-presenting function by metabolic restriction
Source: iScience. 2026 Aug 11;29(8):117144. doi: 10.1016/j.isci.2026.117144 (PMC13487005; doi:10.1016/j.isci.2026.117144)
Supplement: Supplementary file 1 [file mmc1.pdf]

iScience, Volume *29*

Supplemental information

L-asparaginase reversibly suppresses CD40-mediated B cell activation and antigen-presenting function by metabolic restriction

Amar Hadzic, María Alejandra García-Márquez, Pol Bannasch, Nina Haindl, Katharina Frey, Martin Kirmaier, Maximilian A. Funk, Anneli Tischmacher, Jingke Tu, Adriano Carboniero, Ludovica Vona, Sabine Oganesian, Lena Muschik, Hans Schlößer, Martin Vaeth, Werner Schmitz, Michael von Bergwelt-Baildon, David M. Cordas dos Santos, and Sebastian Theurich

Supplementary Information

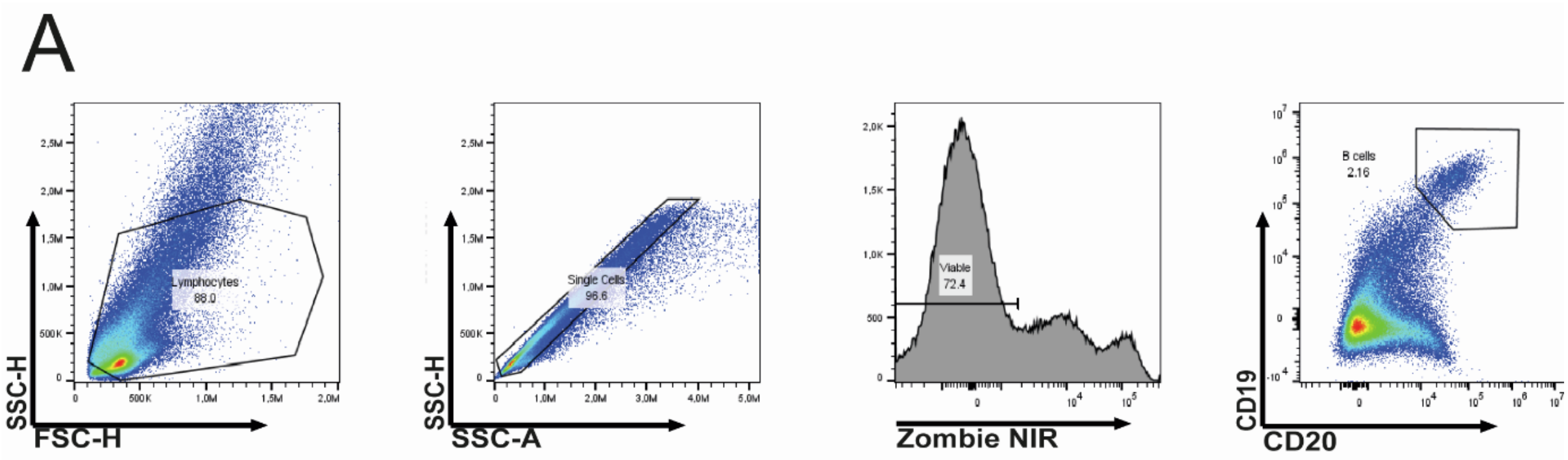

Supplemental Fig. 1: Gating strategy. Flow cytometry samples were gated on lymphocytes using forward and sideward scatter, then single cells using sideward scatter area against height, then on viable Zombie^{-} cells, then on CD19^{+} CD20^{+} B cells.

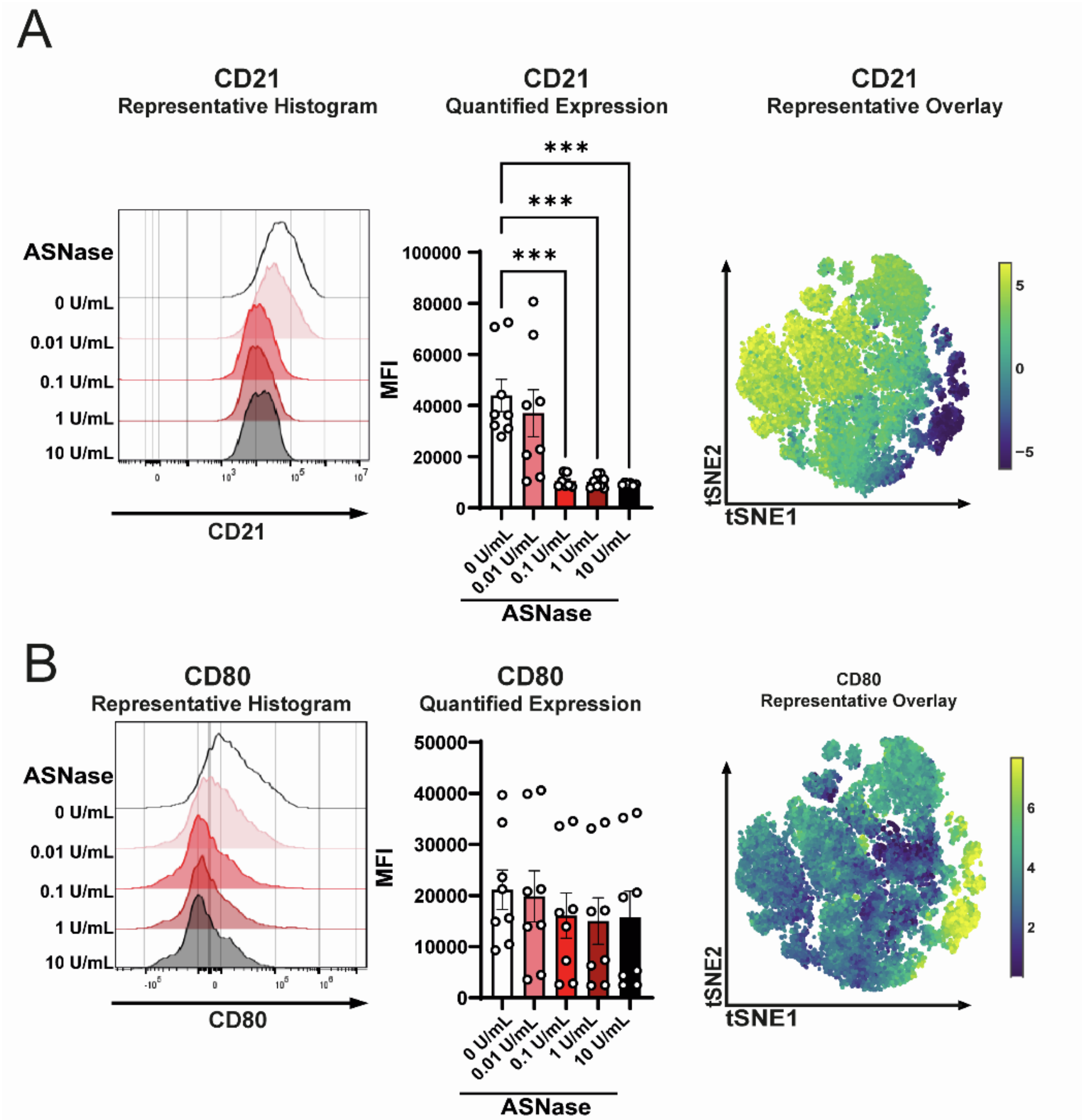

Supplemental Fig. 2: ASNase treatment hinders B cell activation and hinders APC function. Non-malignant donor PBMCs were cultivated for 5 days and activated with CD40L multimer. Cultivation included increasing ASNase concentrations. **A** Representative histogram of CD21 expression on CD19$^+$ CD20$^+$ B cells. Quantitative analysis of CD21 MFIs. Representative overlay of CD21 expression on treated CD19$^+$ CD20$^+$ B cells. **C** Representative histogram of CD80 expression on CD19$^+$ CD20$^+$ B cells. Quantitative analysis of CD80 MFIs. Representative overlay of CD80 expression on treated CD19$^+$ CD20$^+$ B cells. All experiments were performed in biological triplicates or quadruplicates, and biological

replicates were not pooled. Data are represented as mean +/- SEM. Statistical significance was calculated with one-way ANOVA (reference 0 U/ml). Not significant (ns) $P > .05$; *$P < .05$; **$P < .01$; ***$P < .001$; ****$P \leq 0.0001$.

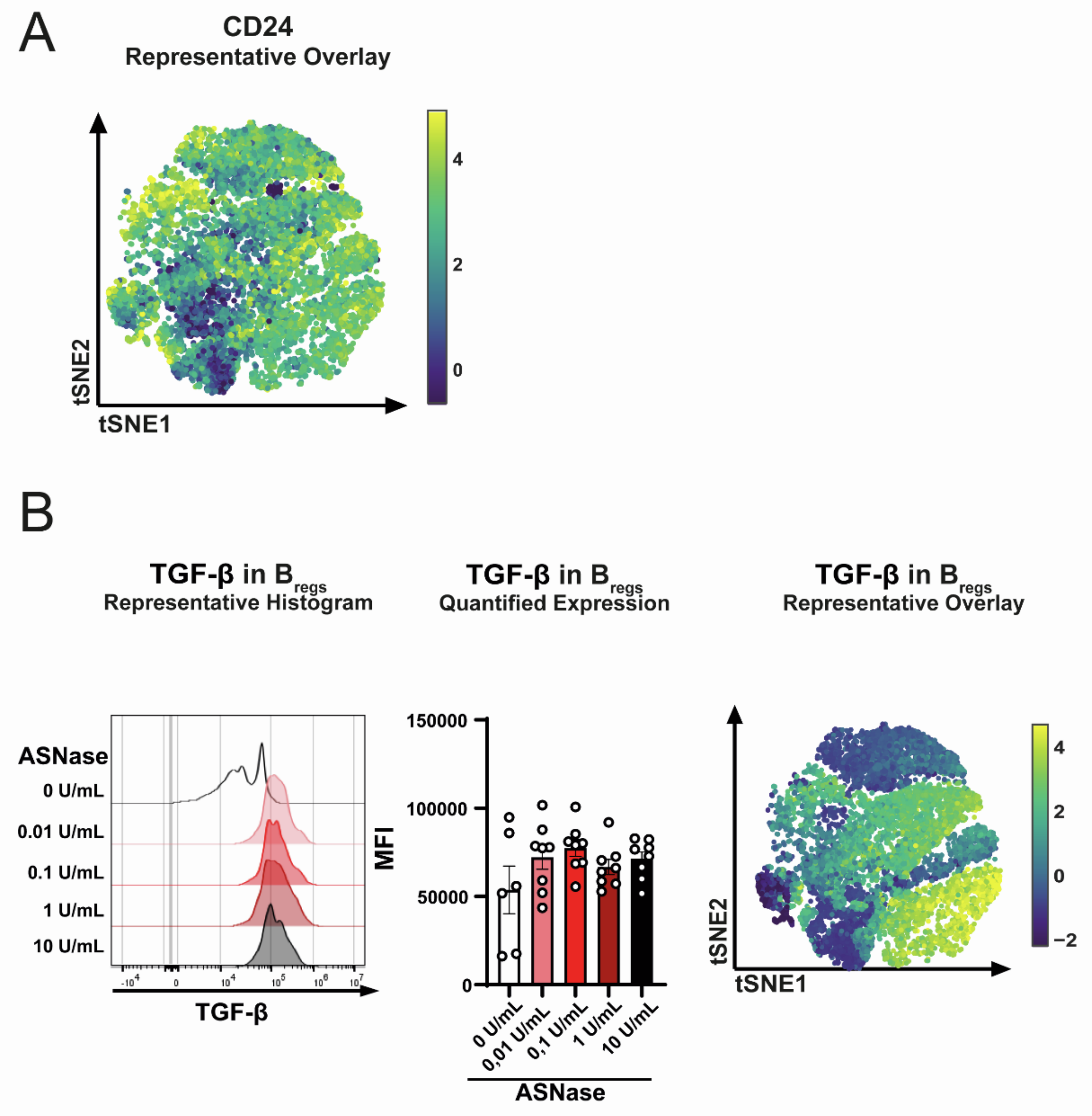
A
CD24
Representative Overlay
tSNE2
tSNE1
4
2
0
B
TGF-β in Bregs
Representative Histogram
ASNase
0 U/mL
0.01 U/mL
0.1 U/mL
1 U/mL
10 U/mL
TGF-β
TGF-β in Bregs
Quantified Expression
150000
100000
50000
0
MFI
0 U/mL
0,01 U/mL
0,1 U/mL
1 U/mL
10 U/mL
ASNase
TGF-β in Bregs
Representative Overlay
tSNE2
tSNE1
4
2
0
−2

Supplemental Fig. 3: ASNase treatment induces Breg formation in a dose-dependent manner. Non-malignant donor PBMCs were cultivated for 5 days and activated with CD40L multimer. Cultivation included increasing ASNase concentrations. **A** Representative overlay of CD24 expression on treated $CD19^{+}$ $CD20^{+}$ B cells. **B** Representative histogram of TGFß expression on $CD19^{+}$ $CD20^{+}$ B cells. Quantitative analysis of TGF-ß MFI. Representative overlay of TGFß expression on treated $CD19^{+}$ $CD20^{+}$ B cells. All experiments were performed in biological triplicates or quadruplicates, and biological replicates were not pooled. Data are represented as mean +/- SEM. Statistical significance was calculated with one-way ANOVA (reference 0 U/ml). Not significant (ns) $P > .05$; $^{*}P < .05$; $^{**}P < .01$; $^{***}P < .001$; $^{****}P \leq 0.0001$.

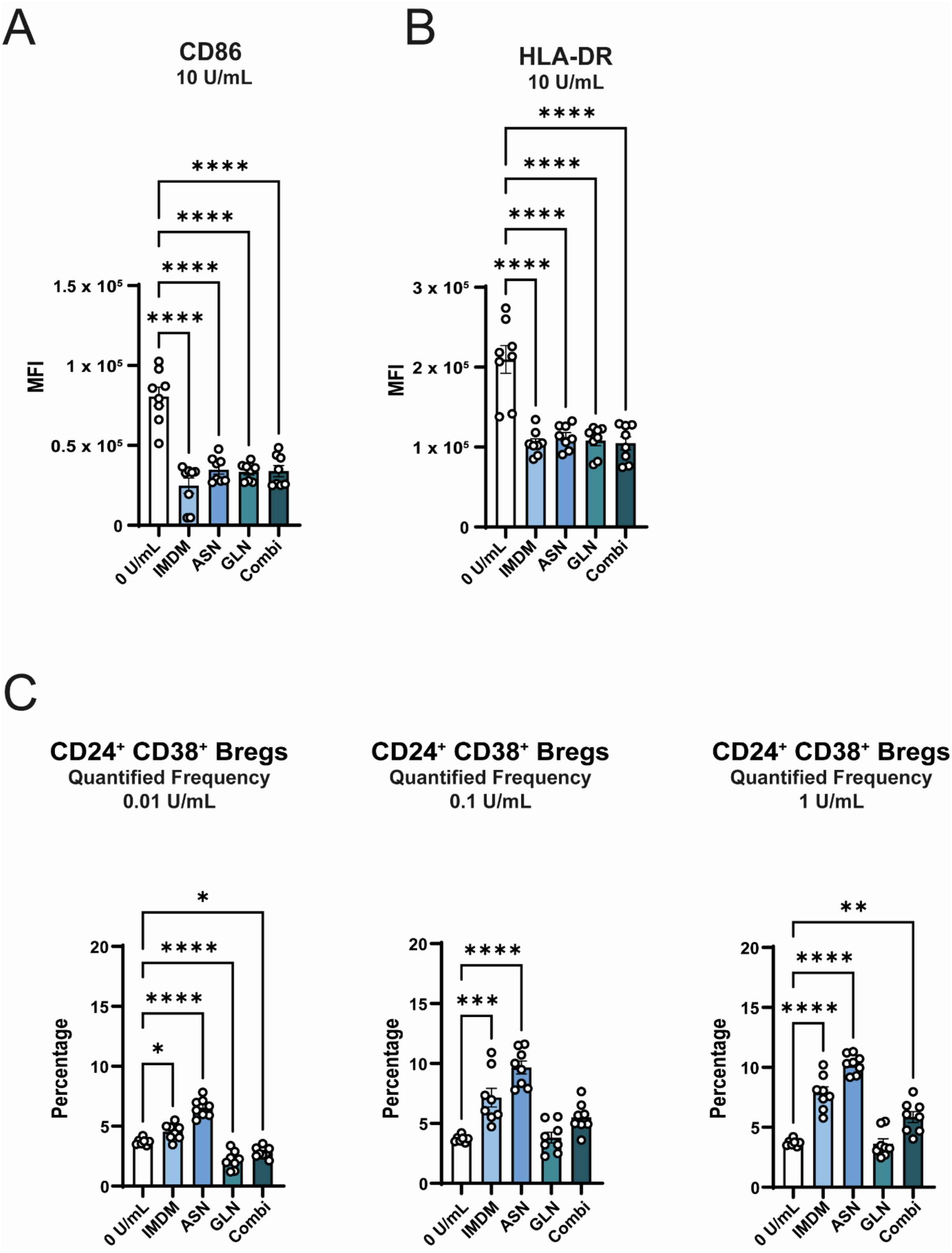

A
CD86
10 U/mL
MFI
1.5 x 10⁵
1 x 10⁵
0.5 x 10⁵
0
0 U/mL
IMDM
ASN
GLN
Combi
****
B
HLA-DR
10 U/mL
3 x 10⁵
2 x 10⁵
1 x 10⁵
C
CD24⁺ CD38⁺ Bregs
Quantified Frequency
0.01 U/mL
0.1 U/mL
1 U/mL
Percentage
20
15
10
5
*
**
***

Supplemental Fig. 4: Exogenous supply of glutamine and / or glutamine rescues B cell APC function and counteracts Breg development. Non-malignant donor PBMCs were cultivated for 5 days and activated with CD40L multimer. Cultivation included increasing ASNase concentrations. **A** Quantitative analysis of CD86 MFI in different media under 10 U/mL ASNase. **B** Quantitative analysis of HLA-DR MFI in different media under 10 U/mL ASNase. **C** Quantification of $CD24^{+}$ and $CD38^{+}$ Bregs in different media under rising ASNase concentrations. All experiments were performed in biological triplicates or quadruplicates, and biological replicates were not pooled. Data are represented as mean +/- SEM. Statistical significance was calculated with one-way ANOVA (reference 0 U/ml). Not significant (ns) $P > .05$; $*P < .05$; $**P < .01$; $***P < .001$; $****P \leq 0.0001$.

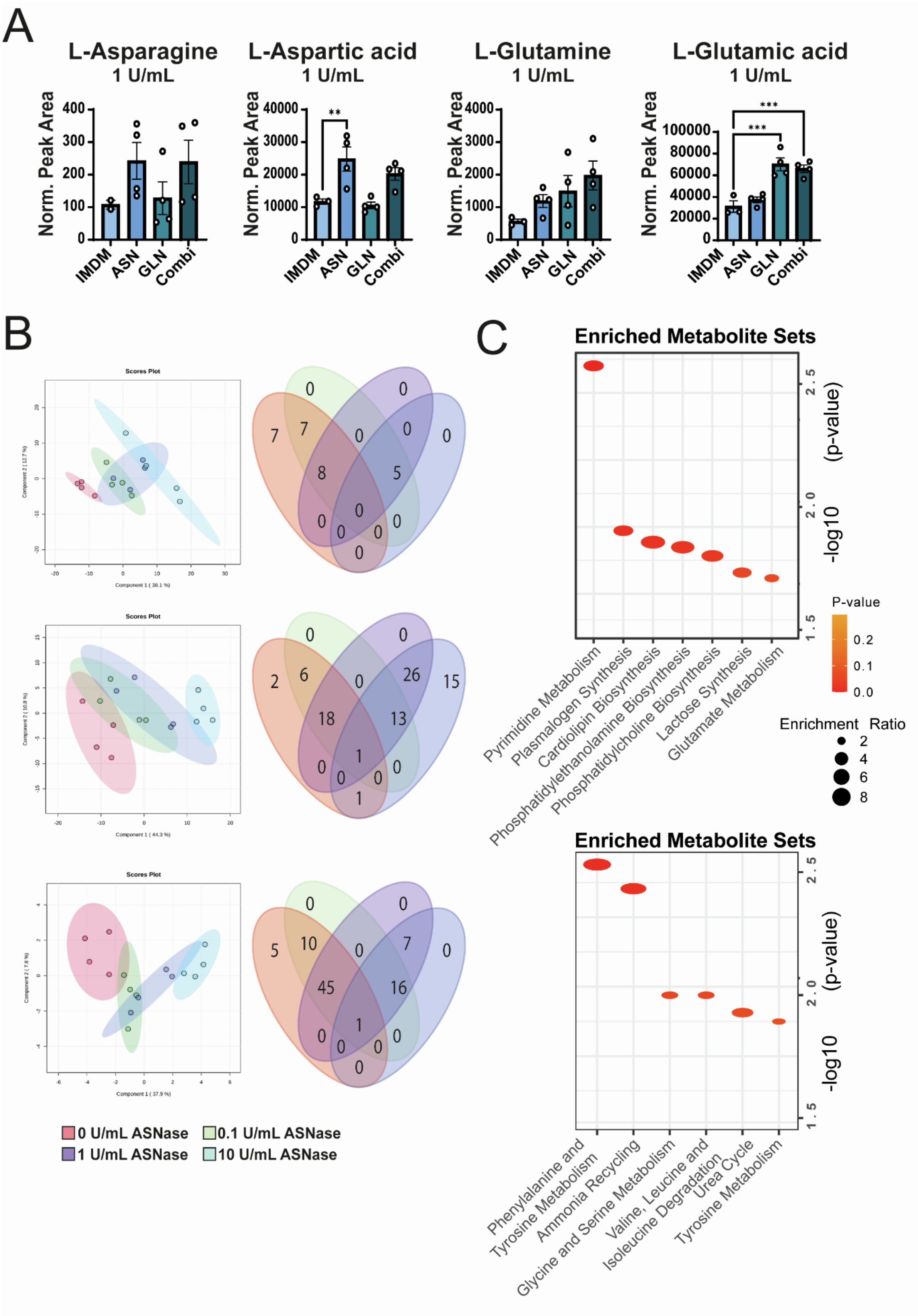
A
L-Asparagine
1 U/mL
L-Aspartic acid
1 U/mL
L-Glutamine
1 U/mL
L-Glutamic acid
1 U/mL
Norm. Peak Area
IMDM
ASN
GLN
Combi
B
Scores Plot
0 U/mL ASNase
0.1 U/mL ASNase
1 U/mL ASNase
10 U/mL ASNase
C
Enriched Metabolite Sets
-log10 (p-value)
Pyrimidine Metabolism
Plasmalogen Synthesis
Cardiolipin Biosynthesis
Phosphatidylethanolamine Biosynthesis
Phosphatidylcholine Biosynthesis
Lactose Synthesis
Glutamate Metabolism
P-value
Enrichment Ratio
Enriched Metabolite Sets
Phenylalanine and Tyrosine Metabolism
Ammonia Recycling
Glycine and Serine Metabolism
Valine, Leucine and Isoleucine Degradation
Urea Cycle
Tyrosine Metabolism

Supplemental Fig. 5: Exogenous supply of asparagine and / or readjusts metabolic profile. Non-malignant donor PBMCs were cultivated for 5 days and activated with CD40L multimer. Cultivation included increasing ASNase concentrations. **A** Normalized peak area of detected L-asparagine, L-aspartic acid, L-glutamine, L-glutamic acid detected in ASNase-treated cells (1 U/mL) and cultivated in supplemented media. **B** PL-SDA plots depicting samples treated different ASNase concentrations in the respective medium and venn-diagramm showing ANOVA testing results for observed metabolites. **C** Enriched metabolite sets based on overrepresented metabolites found in combination media (circled in **Fig. 8B**). All experiments were performed in biological triplicates or quadruplicates, and biological replicates were not pooled. Data are represented as mean +/- SEM. Statistical significance was calculated with one-way ANOVA (reference 0 U/ml). Not significant (ns) $P > .05$; *$P < .05$; **$P < .01$; ***$P < .001$; ****$P \leq 0.0001$.

	0	0.1	1	10
**Cystathionine**	Cystathionine	Cystathionine		
**Uridine diphosphate-N-acetylglucosamine**	Uridine diphosphate-N-acetylglucosamine	Uridine diphosphate-N-acetylglucosamine	Uridine diphosphate-N-acetylglucosamine	
**Carbamoylaspartate**	Carbamoylaspartate (neg.)	Carbamoylaspartate (neg.)		
**dCMP**	dCMP	dCMP		
**GluPro**	GluPro	GluPro		
**LPI(18:1)**	LPI(18:1)	LPI(18:1)		
**Thymidine**	Thymidine	Thymidine		
**L-Valine**		L-Valine	L-Valine	L-Valine
**L-Leucine**		L-Leucine	L-Leucine	L-Leucine
**N-Ac-Met**	N-Ac-Met	N-Ac-Met		
**LPI(20:4)**	LPI(20:4)	LPI(20:4)		
**NAD**	NAD	NAD	NAD	
**NANA**	NANA	NANA		
**Glyceral/-on-P**	Glyceral/-on-P	Glyceral/-on-P		
**Erythritol**	Erythritol	Erythritol		
**Phosphorylcholine**	Phosphorylcholine	Phosphorylcholine		
**ArgSucc**	ArgSucc	ArgSucc		
**GlcNAc-P**	GlcNAc-P	GlcNAc-P	GlcNAc-P	
**Putrescine**	Putrescine	Putrescine		
**AC-(04:0)**	AC-(04:0)	AC-(04:0)		
**Phenyllactate**	Phenyllactate	Phenyllactate		
**Uridine 5'-monophosphate**	Uridine 5'-monophosphate	Uridine 5'-monophosphate	Uridine 5'-monophosphate	
**Cytidine monophosphate**	Cytidine monophosphate	Cytidine monophosphate		
**L-Aspartic acid**		L-Aspartic acid	L-Aspartic acid	L-Aspartic acid
**GSH**	GSH	GSH		
**Adenosine monophosphate**	Adenosine monophosphate	Adenosine monophosphate		
**Oxidized glutathione**	Oxidized glutathione	Oxidized glutathione		
**L-Asparagine**	L-Asparagine			
**CMP-Sialic Acid**	CMP-Sialic Acid	CMP-Sialic Acid		
**AC-(03:0)**	AC-(03:0)	AC-(03:0)		
**Deoxyuridine**	Deoxyuridine	Deoxyuridine		
**L-Phenylalanine**			L-Phenylalanine	L-Phenylalanine
**Uridine diphosphate glucose**	Uridine diphosphate glucose	Uridine diphosphate glucose		
**Phosphocreatine**	Phosphocreatine	Phosphocreatine		
**L-Methionine**		L-Methionine	L-Methionine	L-Methionine
**Guanosine monophosphate**	Guanosine monophosphate	Guanosine monophosphate		

**Phosphoenolpyruvic acid**	Phosphoenolpyruvic acid	Phosphoenolpyruvic acid		
**Methylthioadenosine**	Methylthioadenosine	Methylthioadenosine		
**GPI**	GPI	GPI		
**L-Alanine**	L-Alanine	L-Alanine		
**Mevalonate-5-P**	Mevalonate-5-P			
**Serine**		Serine	Serine	Serine
**Aldohexose**		Aldohexose	Aldohexose	Aldohexose
**Bisphosphoglycerate**	Bisphosphoglycerate	Bisphosphoglycerate		
**Uridine diphosphate glucuronic acid**	Uridine diphosphate glucuronic acid			
**Orotic acid**	Orotic acid	Orotic acid		
**Glucosamine-P**	Glucosamine-P	Glucosamine-P		
**GPS**	GPS	GPS		
**XMP**	XMP	XMP		

Supplemental Table 1: Enriched metabolites in ASNase treated cells. Non-malignant donor PBMCs were cultivated for 5 days and activated with CD40L multimer. Cultivation included increasing ASNase concentrations. Metabolomics concluded as described and analysis performed using Metaboanalyst 6.0 ANOVA testing. P values ≤ 0.05 were considered statistically significant.

	0	0.1	1	10
**LPI(20:3)**	LPI(20:3)			
**N5-Methyl-FH4**	N5-Methyl-FH4			
**FAD**	FAD	FAD	FAD	
**5-Thymidylic acid**	5-Thymidylic acid	5-Thymidylic acid		
**Thymidine**	Thymidine	Thymidine	Thymidine	
**AC-(04:0)**	AC-(04:0)	AC-(04:0)	AC-(04:0)	
**N-Ac-Met**	N-Ac-Met	N-Ac-Met	N-Ac-Met	
**Acetoacetic acid**		Acetoacetic acid	Acetoacetic acid	Acetoacetic acid
**Uridine**	Uridine			
**Cytosine**	Cytosine			
**Aldohexose**		Aldohexose	Aldohexose	Aldohexose
**L-Asparagine**	L-Asparagine			
**Cytidine**	Cytidine	Cytidine		
**Serine**		Serine	Serine	Serine
**Phenyllactate**	Phenyllactate	Phenyllactate	Phenyllactate	
**Uridine 5'-monophosphate**	Uridine 5'-monophosphate	Uridine 5'-monophosphate		
**LPI(20:4)**	LPI(20:4)	LPI(20:4)		
**dRibose-P**		dRibose-P	dRibose-P	dRibose-P
**Deoxyuridine**	Deoxyuridine	Deoxyuridine		
**CMP-Sialic Acid**	CMP-Sialic Acid	CMP-Sialic Acid	CMP-Sialic Acid	
**Erythritol**	Erythritol	Erythritol	Erythritol	
**L-Carnitine**	L-Carnitine	L-Carnitine		
**Orotic acid**	Orotic acid			
**XMP**	XMP	XMP		
**LPI(18:1)**	LPI(18:1)			
**N-Ac-Glu**	N-Ac-Glu	N-Ac-Glu	N-Ac-Glu	

Supplemental Table 2: Enriched metabolites in ASNase treated cells in asn-supplemented medium. Non-malignant donor PBMCs were cultivated for 5 days and activated with CD40L multimer. Cultivation included increasing ASNase concentrations. Metabolomics concluded as described and analysis performed using Metaboanalyst 6.0 ANOVA testing. P values ≤ 0.05 were considered statistically significant.

	0	0.1	1	10
**L-Lysine**		L-Lysine	L-Lysine	L-Lysine
**Pyruvic acid**		Pyruvic acid	Pyruvic acid	Pyruvic acid
**Methioninesulfoxide**			Methioninesulfoxide	Methioninesulfoxide
**GlcNAc-sulfate**	GlcNAc-sulfate	GlcNAc-sulfate	GlcNAc-sulfate	
**L-Glutamic acid**		L-Glutamic acid	L-Glutamic acid	L-Glutamic acid
**2,3-Diketogulonic Acid**		2,3-Diketogulonic Acid	2,3-Diketogulonic Acid	2,3-Diketogulonic Acid
**Serine**		Serine	Serine	Serine
**3-Hydroxyvaleric acid**			3-Hydroxyvaleric acid	3-Hydroxyvaleric acid
**Phenylpyruvate**		Phenylpyruvate	Phenylpyruvate	Phenylpyruvate
**Acetoacetic acid**			Acetoacetic acid	Acetoacetic acid
**Cysteinesulfate**			Cysteinesulfate	Cysteinesulfate
**Tetrahydrobiopterin**	Tetrahydrobiopterin	Tetrahydrobiopterin	Tetrahydrobiopterin	
**Spermidine**			Spermidine	Spermidine
**L-Valine**			L-Valine	L-Valine
**L-Tyrosine**			L-Tyrosine	L-Tyrosine
**dRibose-P**			dRibose-P	dRibose-P
**Aldohexose**		Aldohexose	Aldohexose	Aldohexose
**L-Leucine**			L-Leucine	L-Leucine
**Pipecolate**			Pipecolate	Pipecolate
**S-Adenosylhomocysteine**	S-Adenosylhomocysteine	S-Adenosylhomocysteine	S-Adenosylhomocysteine	
**CMP-Sialic Acid**	CMP-Sialic Acid	CMP-Sialic Acid	CMP-Sialic Acid	
**dUDP**	dUDP	dUDP	dUDP	
**L-Asparagine**	L-Asparagine	L-Asparagine	L-Asparagine	
**L-Isoleucine**			L-Isoleucine	L-Isoleucine
**L-Arginine**			L-Arginine	L-Arginine
**L-Cystine**			L-Cystine	L-Cystine
**Uridine diphosphate-N-acetylglucosamine**	Uridine diphosphate-N-acetylglucosamine	Uridine diphosphate-N-acetylglucosamine	Uridine diphosphate-N-acetylglucosamine	
**2-Methylbutyrylglycine**				2-Methylbutyrylglycine
**L-Alanine**		L-Alanine	L-Alanine	L-Alanine
**Alpha-ketoisovaleric acid**		Alpha-ketoisovaleric acid	Alpha-ketoisovaleric acid	Alpha-ketoisovaleric acid
**Choline**			Choline	Choline
**L-Phenylalanine**			L-Phenylalanine	L-Phenylalanine
**Pantothenic acid**			Pantothenic acid	Pantothenic acid
**Homogentisate**				Homogentisate
**L-Threonine**		L-Threonine	L-Threonine	L-Threonine
**Creatinine**			Creatinine	Creatinine
**Urocanic acid**				Urocanic acid
**Folic acid**			Folic acid	Folic acid

**L-Methionine**			L-Methionine	L-Methionine
**Glycine**		Glycine	Glycine	Glycine
**4-Hydroxyphenylpyruvic acid**				4-Hydroxyphenylpyruvic acid
**LPI(20:3)**	LPI(20:3)			
**L-Aspartic acid**		L-Aspartic acid	L-Aspartic acid	L-Aspartic acid
**L-Histidine**			L-Histidine	L-Histidine
**O-Phosphoethanolamine**		O-Phosphoethanolamine	O-Phosphoethanolamine	O-Phosphoethanolamine
**Glycerol 3-phosphate**	Glycerol 3-phosphate	Glycerol 3-phosphate	Glycerol 3-phosphate	
**ArgSucc**	ArgSucc	ArgSucc	ArgSucc	
**XMP**	XMP	XMP	XMP	
**3-Hydroxybutyrate**			3-Hydroxybutyrate	3-Hydroxybutyrate
**AC-(08:1)**			AC-(08:1)	AC-(08:1)
**Thymine**				Thymine
**Pyridoxine**			Pyridoxine	Pyridoxine
**Guanidinosuccinic acid**				Guanidinosuccinic acid
**5-Hydroxyindoleacetic acid**			5-Hydroxyindoleacetic acid	5-Hydroxyindoleacetic acid
**GlcNAc-P**	GlcNAc-P	GlcNAc-P	GlcNAc-P	
**Orotic acid**	Orotic acid	Orotic acid		
**GPI**	GPI	GPI		
**5-Hydroxylysine**				5-Hydroxylysine
**LPI(20:4)**	LPI(20:4)	LPI(20:4)		
**2-Hydroxybutyrate**				2-Hydroxybutyrate
**CDP-Ethanolamine**	CDP-Ethanolamine	CDP-Ethanolamine	CDP-Ethanolamine	
**Uridine diphosphate glucose**	Uridine diphosphate glucose	Uridine diphosphate glucose	Uridine diphosphate glucose	
**Citrulline**				Citrulline
**Citric acid**				Citric acid
**D-Sedoheptulose 7-phosphate**	D-Sedoheptulose 7-phosphate	D-Sedoheptulose 7-phosphate	D-Sedoheptulose 7-phosphate	
**Ascorbate**			Ascorbate	Ascorbate
**1-Methylhistidine**				1-Methylhistidine
**Cytidine**	Cytidine	Cytidine		
**NANA**	NANA (neg.)	NANA (neg.)	NANA (neg.)	
**Dimethylglycine**				Dimethylglycine
**L-Tryptophan**			L-Tryptophan	L-Tryptophan
**Aminoadipic acid**				Aminoadipic acid
**GluAsn**	GluAsn	GluAsn	GluAsn	
**Oxoglutaric acid**				Oxoglutaric acid
**Carbamoylaspartate**	Carbamoylaspartate (neg.)	Carbamoylaspartate (neg.)		

**L-Glutamine**	L-Glutamine			
**Thymidine**	Thymidine	Thymidine		
**GluAsp**	GluAsp	GluAsp	GluAsp	
**Uridine 5'-monophosphate**	Uridine 5'-monophosphate	Uridine 5'-monophosphate	Uridine 5'-monophosphate	
**Ribitol**				Ribitol

Supplemental Table 3: Enriched metabolites in ASNase treated cells in gln-supplemented medium. Non-malignant donor PBMCs were cultivated for 5 days and activated with CD40L multimer. Cultivation included increasing ASNase concentrations. Metabolomics concluded as described and analysis performed using Metaboanalyst 6.0 ANOVA testing. P values ≤ 0.05 were considered statistically significant.

	0	0.1	1	10
**Acetoacetic acid**		Acetoacetic acid	Acetoacetic acid	Acetoacetic acid
**Hexose-P**	Hexose-P	Hexose-P	Hexose-P	
**Guanosine monophosphate**	Guanosine monophosphate	Guanosine monophosphate	Guanosine monophosphate	
**D-Sedoheptulose 7-phosphate**	D-Sedoheptulose 7-phosphate	D-Sedoheptulose 7-phosphate	D-Sedoheptulose 7-phosphate	
**Adenosine monophosphate**	Adenosine monophosphate	Adenosine monophosphate	Adenosine monophosphate	
**CMP-Sialic Acid**	CMP-Sialic Acid	CMP-Sialic Acid	CMP-Sialic Acid	
**Orotic acid**	Orotic acid	Orotic acid	Orotic acid	
**Cytidine monophosphate**	Cytidine monophosphate	Cytidine monophosphate	Cytidine monophosphate	
**Uridine 5'-monophosphate**	Uridine 5'-monophosphate	Uridine 5'-monophosphate	Uridine 5'-monophosphate	
**L-Valine**		L-Valine	L-Valine	L-Valine
**GPI**	GPI	GPI	GPI	
**S-Adenosylhomocysteine**	S-Adenosylhomocysteine	S-Adenosylhomocysteine	S-Adenosylhomocysteine	
**Adenylsuccinic acid**	Adenylsuccinic acid	Adenylsuccinic acid	Adenylsuccinic acid	
**Aldohexose**		Aldohexose	Aldohexose	Aldohexose
**L-Tyrosine**		L-Tyrosine	L-Tyrosine	L-Tyrosine
**Cytidine**	Cytidine	Cytidine	Cytidine	
**Dimethylarginine**	Dimethylarginine	Dimethylarginine	Dimethylarginine	
**CDP-Ethanolamine**	CDP-Ethanolamine	CDP-Ethanolamine	CDP-Ethanolamine	
**Carbamoylaspartate**	Carbamoylaspartate	Carbamoylaspartate	Carbamoylaspartate	
**L-Cystine**			L-Cystine	L-Cystine
**5-Thymidylic acid**	5-Thymidylic acid	5-Thymidylic acid	5-Thymidylic acid	
**XMP**	XMP	XMP	XMP	
**Pyruvic acid**		Pyruvic acid	Pyruvic acid	Pyruvic acid
**L-Aspartic acid**		L-Aspartic acid	L-Aspartic acid	L-Aspartic acid
**N-Ac-Met**	N-Ac-Met	N-Ac-Met	N-Ac-Met	
**Glucosamine-P**	Glucosamine-P	Glucosamine-P	Glucosamine-P	
**GPS**	GPS	GPS	GPS	
**ArgSucc**	ArgSucc	ArgSucc	ArgSucc	
**dRibose-P**		dRibose-P	dRibose-P	dRibose-P
**IMP**	IMP	IMP	IMP	
**Pyroglutamic acid**	Pyroglutamic acid	Pyroglutamic acid		
**L-Lysine**		L-Lysine	L-Lysine	L-Lysine
**Uridine diphosphate-N-acetylglucosamine**	Uridine diphosphate-N-acetylglucosamine	Uridine diphosphate-N-acetylglucosamine	Uridine diphosphate-N-acetylglucosamine	
**Erythritol**	Erythritol	Erythritol	Erythritol	
**Serine**		Serine	Serine	Serine

**Thiamine monophosphate**	Thiamine monophosphate	Thiamine monophosphate	Thiamine monophosphate	
**Phosphorylcholine**	Phosphorylcholine	Phosphorylcholine	Phosphorylcholine	
**Uracil**	Uracil			
**Uridine**	Uridine			
**Choline**			Choline	Choline
**dAMP**	dAMP	dAMP		
**L-Methionine**		L-Methionine	L-Methionine	L-Methionine
**NADPH**	NADPH	NADPH	NADPH	
**GluAsp**	GluAsp	GluAsp	GluAsp	
**Uridine diphosphate glucose**	Uridine diphosphate glucose	Uridine diphosphate glucose	Uridine diphosphate glucose	
**Glycerylphosphoryl ethanolamine**	Glycerylphosphoryleth anolamine	Glycerylphosphoryleth anolamine	Glycerylphosphoryleth anolamine	
**GluAsn**	GluAsn	GluAsn	GluAsn	
**LPI(20:3)**	LPI(20:3)			
**Thymidine**	Thymidine	Thymidine		
**N-Ac-Glu**	N-Ac-Glu	N-Ac-Glu	N-Ac-Glu	
**L-Asparagine**	L-Asparagine			
**Biotin**	Biotin	Biotin	Biotin	
**GlcNAc-P**	GlcNAc-P	GlcNAc-P	GlcNAc-P	
**FAD**	FAD	FAD	FAD	
**Tetrahydrobiopterin**	Tetrahydrobiopterin	Tetrahydrobiopterin	Tetrahydrobiopterin	
**Cytosine**	Cytosine	Cytosine		
**Folic acid**		Folic acid	Folic acid	Folic acid
**Phenylpyruvate**		Phenylpyruvate	Phenylpyruvate	Phenylpyruvate
**Biopterin**	Biopterin	Biopterin	Biopterin	
**dUDP**	dUDP (neg.)	dUDP (neg.)	dUDP (neg.)	
**Glycerol 3-phosphate**	Glycerol 3-phosphate	Glycerol 3-phosphate	Glycerol 3-phosphate	
**AC-(06:0)**	AC-(06:0)	AC-(06:0)		
**AC-(08:1)**		AC-(08:1)	AC-(08:1)	AC-(08:1)
**L-Histidine**			L-Histidine	L-Histidine
**L-Leucine**		L-Leucine	L-Leucine	L-Leucine
**L-Glutamine**	L-Glutamine	L-Glutamine		
**Methioninesulfoxide**			Methioninesulfoxide	Methioninesulfoxide
**LPI(20:4)**	LPI(20:4)	LPI(20:4)		
**NANA**	NANA	NANA	NANA	
**Phosphocreatine**	Phosphocreatine	Phosphocreatine	Phosphocreatine	
**Deoxyuridine**	Deoxyuridine	Deoxyuridine		
**Phosphoenolpyruvic acid**	Phosphoenolpyruvic acid	Phosphoenolpyruvic acid	Phosphoenolpyruvic acid	
**L-Arginine**			L-Arginine	L-Arginine
**Pentose 5-phosphate**	Pentose 5-phosphate	Pentose 5-phosphate	Pentose 5-phosphate	
**GluPro**	GluPro	GluPro	GluPro	

**L-Malic acid**	L-Malic acid	L-Malic acid		
**dCMP**	dCMP (pos.)	dCMP (pos.)		
**2,3-Diketogulonic Acid**		2,3-Diketogulonic Acid	2,3-Diketogulonic Acid	2,3-Diketogulonic Acid
**GluGlu**	GluGlu			
**Creatinine**			Creatinine	Creatinine
**L-Isoleucine**			L-Isoleucine	L-Isoleucine
**5-Hydroxyindoleacetic acid**		5-Hydroxyindoleacetic acid	5-Hydroxyindoleacetic acid	5-Hydroxyindoleacetic acid
**Taurine**	Taurine	Taurine	Taurine	

Supplemental Table 4: Enriched metabolites in ASNase treated cells in asn and gln supplemented medium. Non-malignant donor PBMCs were cultivated for 5 days and activated with CD40L multimer. Cultivation included increasing ASNase concentrations. Metabolomics concluded as described and analysis performed using Metaboanalyst 6.0 ANOVA testing. P values ≤ 0.05 were considered statistically significant.
